# The fibroblast Tiam1-osteopontin pathway modulates breast cancer invasion and metastasis

**DOI:** 10.1186/s13058-016-0674-8

**Published:** 2016-01-28

**Authors:** Kun Xu, Xuejun Tian, Sun Y. Oh, Mohammad Movassaghi, Stephen P. Naber, Charlotte Kuperwasser, Rachel J. Buchsbaum

**Affiliations:** Molecular Oncology Research Institute, Tufts Medical Center, 75 Kneeland Street, Boston, MA 02111 USA; Department of Pathology, Tufts Medical Center, 800 Washington Street, Boston, MA 02111 USA; Department of Medicine, Tufts Medical Center, 800 Washington Street, Boston, MA 02111 USA; Department of Chemistry, Massachusetts Institute of Technology, 77 Massachusetts Avenue, Cambridge, MA 02139 USA; Developmental, Molecular, and Chemical Biology Department, Tufts University School of Medicine, 136 Harrison Avenue, Boston, MA 02111 USA

**Keywords:** Tiam1, Osteopontin, Breast cancer, Cancer-associated fibroblast, Agelastatin, Metastasis

## Abstract

**Background:**

The tumor microenvironment has complex effects in cancer pathophysiology that are not fully understood. Most cancer therapies are directed against malignant cells specifically, leaving pro-malignant signals from the microenvironment unaddressed. Defining specific mechanisms by which the tumor microenvironment contributes to breast cancer metastasis may lead to new therapeutic approaches against advanced breast cancer.

**Methods:**

We use a novel method for manipulating three-dimensional mixed cell co-cultures, along with studies in mouse xenograft models of human breast cancer and a histologic study of human breast cancer samples, to investigate how breast cancer-associated fibroblasts affect the malignant behaviors of breast cancer cells.

**Results:**

Altering fibroblast Tiam1 expression induces changes in invasion, migration, epithelial-mesenchymal transition, and cancer stem cell characteristics in associated breast cancer cells. These changes are both dependent on fibroblast secretion of osteopontin and also long-lasting even after cancer cell dissociation from the fibroblasts, indicating a novel Tiam1-osteopontin pathway in breast cancer-associated fibroblasts. Notably, inhibition of fibroblast osteopontin with low doses of a novel small molecule prevents lung metastasis in a mouse model of human breast cancer metastasis. Moreover, fibroblast expression patterns of Tiam1 and osteopontin in human breast cancers show converse changes correlating with invasion, supporting the hypothesis that this pathway in tumor-associated fibroblasts regulates breast cancer invasiveness in human disease and is thus clinically relevant.

**Conclusions:**

These findings suggest a new therapeutic paradigm for preventing breast cancer metastasis. Pro-malignant signals from the tumor microenvironment with long-lasting effects on associated cancer cells may perpetuate the metastatic potential of developing cancers. Inhibition of these microenvironment signals represents a new therapeutic strategy against cancer metastasis that enables targeting of stromal cells with less genetic plasticity than associated cancer cells and opens new avenues for investigation of novel therapeutic targets and agents.

**Electronic supplementary material:**

The online version of this article (doi:10.1186/s13058-016-0674-8) contains supplementary material, which is available to authorized users.

## Background

The tumor microenvironment plays an important role in tumor evolution and the reciprocal signals between cancers and the surrounding microenvironments are beginning to be elucidated [1, 2]. There is growing evidence that the stromal microenvironment around cancer cells influences the growth, invasiveness, and metastatic behavior of cancer cells, and may be a factor in therapeutic response. Tumor-associated stroma contains various cell types and molecules secreted into the extracellular matrix, and the list of factors that participate in the co-evolution of tumors with tumor-associated stroma is growing [3]. Of note, fibroblasts are the predominant cell type in stromal connective tissue, contributing to deposition and maintenance of collagen, basement membrane and paracrine growth factors. There is emerging evidence that fibroblasts may actively function in the induction of cancers [4], and that tumor invasion is influenced by external signals from the tumor-associated stroma [5, 6]. Specific mechanisms underlying how the tumor microenvironment contributes to breast cancer metastasis are not yet fully understood.

We have investigated whether the Rac exchange factor Tiam1 in the human breast cancer microenvironment has a role in regulating tumor invasion and metastasis. This question initially arose from a paradox in understanding Tiam1. Tiam1 is a ubiquitous protein involved in a number of signaling pathways with varied functional outcomes depending on cellular context [7–13]. Much of the work on Tiam1 has focused on its role within cancer cells, and Tiam1 expression in tumor cells is required for facilitating tumor growth [14–17]. However, in mice genetically lacking Tiam1, the tumors that do develop are more invasive, conceptually inconsistent with the requirement for Tiam1 for tumor growth and functionally inconsistent with how human tumors behave clinically. We therefore hypothesized that decreased tumor growth in these mice is due to Tiam1 deficiency in the tumor cells, while the increased tumor invasion is due to Tiam1 deficiency in the tumor stroma, specifically in the fibroblasts. In epithelial cells Tiam1 expression is Wnt-responsive and regulated by post-translational events, including phosphorylation and proteolysis [14, 18–20]. Many groups have investigated Tiam1 expression in different tumor cell models and human tumor specimens [14, 21–27]. However, we have chosen to focus on the role of Tiam1 in tumor-associated stroma. Using three different experimental systems, including three-dimensional (3D) mixed cell spheroid co-cultures of mammary epithelial and fibroblast cells, a 3D organotypic culture model of human skin tumors, and a mouse xenograft model of human breast cancer, we recently reported that Tiam1 deficiency in tumor-associated fibroblasts induces increased invasion of the associated epithelial or tumor cells and increased tumor metastasis [28].

We have also found that the glycoprotein osteopontin (OPN) is a major mediator of the effects of fibroblast Tiam1 expression in fibroblasts undergoing stress-induced senescence [29]. Changes in fibroblast Tiam1 expression induce converse changes in OPN transcription and protein secretion. Stress-induced senescence in fibroblasts induces decreased fibroblast Tiam1 and increased OPN expression, and subsequently increased invasion of co-cultured mammary epithelial cells. OPN is a phosphorylated glycoprotein secreted by multiple cell types, both malignant and non-malignant [30]. It is detectable in extracellular matrix, tissues, and body fluids, and is implicated in several pathophysiologic processes, including tumor progression (reviewed in [31]). We therefore investigated the hypothesis that the Tiam1-OPN pathway in mammary fibroblasts influences the invasive and metastatic behavior of breast cancers.

## Methods

### Cell culture

The parental reduction mammary fibroblast (RMF) fibroblast line and the human breast cancer cell lines were kind gifts from Dr. Charlotte Kuperwasser, Tufts University. Derivation of the stable protein-silencing or protein-expressing sublines has been described previously, as follows:

- Tiam1-deficient shTiam-RMF and retroviral hairpin control RMF (C-RMF) [28];
- Tiam1-overexpressing (+Tiam) RMF, pBabe vector control RMF, Tiam1 and OPN-deficient shTiam-OPN RMF and the double retroviral-lentiviral control [29]; human breast cancer cells SUM1315 and SUM159 [32].

Methods indicated, as previously described, are included in more detail in the supplementary material (see Additional file 1).

### Spheroid co-culture

Co-cultures were established as previously described [33]. Where indicated, the following were incorporated into Matrigel and culture medium: 667 ng/mL anti-OPN antibody (Santa Cruz Biotechnology, Dallas, TX, USA); 667 ng/mL isotypic immunoglobulin G (IgG) control antibody; 75 nM Agelastatin A (AgelA); 750 uM cyclophosphamide; 1 nM docetaxel; 8 nM doxorubicin. AgelA was synthesized as previously described [34]. Stock solutions (1 mM) were stored in methanol at -20 °C.

### Isolation of cells from spheroid co-culture

Cells were isolated from 3D co-cultures and cancer cells were separated from fibroblasts as described previously to derive post-co-culture (PCC) cell populations [33]. Flow cytometry was used to demonstrate purity of cells isolated post-co-culture to greater than 99 % cancer cells using fluorescent markers (RMFs with green fluorescent protein (GFP), cancer cells with or without pCherry).

### Growth curves

RMF or SUM1315 cells were seeded at 3 × 10^5^ cells per 100 cm culture dish in appropriate culture medium in the absence or presence of AgelA at final indicated concentrations, and passed every 2 days (3 × 10^5^ cells per passage). Total number of cells in each dish was calculated as the number of cells in the dish at each time point corrected for the fractional passages.

### Immunoblotting

Immunoblotting was performed as previously described [28]. Antibodies were obtained from the following sources: Tiam, GAPDH, Fibronectin, Twist (Santa Cruz Biotechnology); Snail, Slug, E-cadherin, N-cadherin (Cell Signaling Technology, Danvers, MA, USA).

### Transwell migration assay

Standard transwell migration assays were performed as previously described [29]. For experiments testing the differential effect of OPN antibody against cancer cells compared with fibroblasts, SUM1315 cells were seeded in the top wells, and control hairpin vector (shC) or Tiam1-deficient RMF (shT) cells were seeded in the bottom wells as indicated (2 × 10^4^ cells/well for each cell type). Some SUM1315 were incubated with control IgG or OPN antibody (6.7 μg/mL) for 16 h in culture prior to assay as well as during the 5-h migration assay, as indicated (top well Ab). RMF were seeded into bottom wells 16 h before assay to establish conditioned medium and similarly incubated with indicated antibodies before and during the assay (bottom well Ab).

### Tumorsphere assays

Tumorsphere formation was assessed as previously described [32].

### Flow cytometry

Flow cytometry was performed as previously described [32].

### Real-time PCR

Isolation of ribonucleic acid (RNA) and quantitative reverse transcriptase polymerase chain reaction (RT-PCR) were performed as previously described [29], using the following primer pairs:

- Snail, 5′-AATCGGAAGCCTAACTACAGCG-3′ (sense), 5′-GTCCCAGATGAGCATTGGCA-3′ (antisense);
- Slug, 5′-AAGCATTTCAACGCCTCCAAA-3′ (sense), 5′-AGGATCTCTGGTTGTGGTATGAC-3′ (antisense);
- Twist, 5′-GTCCGCAGTCTTACGAGGAG-3′ (sense), 5′-GCTTGAGGGTCTGAATCTTGCT-3′ (antisense);
- Fibronectin, 5′-CAGTGGGAGACCTCGAGAAG-3′ (sense), 5′-TCCCTCGGAACATCAGAAAC-3′ (antisense);
- N-Cadherin, 5′-TCCTACTGGACGGTTCGCCA-3′ (sense), 5′-TTGCAGTTGACTGAGGCGGG-3′ (antisense);
- E-Cadherin, 5′-CCCACCACGTACAAGGGTC-3′ (sense), 5′-ATGCCATCGTTGTTCACTGGA-3′ (antisense);
- GAPDH, 5′-CTGCACCACCAACTGCTTAG-3′ (sense), 5′-TTCAGCTCAGGGATGACCTT-3′ (antisense);
- Tiam1, 5′- AAGACGTACTCAGGCCATGTCC-3′ (sense), 5′-GACCCAAATGTCGCAGTCAG-3′ (antisense);
- OPN, 5′-GCCATACCAGTTAAACAGGC -3′ (sense), 5′- GACCTCAGAAGATGCACTAT -3′ (antisense).

### Human breast cancer xenograft models

#### Primary tumors

PCC-SUM1315 cells were isolated from co-cultures with control, Tiam1-deficient, or Tiam1-overexpressing fibroblasts as indicated. PCC-non-fluorescing SUM1315 cells were further purified from GFP-expressing RMFs by flow cytometry, suspended in a mixture of Matrigel and SUM1315 culture medium (1:3 ratio), and injected into fourth inguinal mammary glands in a 35 μL volume into NOD-SCID mice (The Jackson Laboratory, Bar Harbor, ME, USA).

Passaged tumors were established as previously described [32].

### Immunohistochemical staining of human tumor samples

Immunohistochemistry was performed on the Ventana Benchmark XT (Ventana Medical Systems, Inc., Tucson, AZ, USA) using the following antibodies: Tiam1, Santa Cruz sc-872; osteopontin, Abcam (Cambridge, MA, USA) ab33046. Assessment of Tiam1 and osteopontin staining in human breast cancer tissue was performed by two board-certified pathologists, one of whom is an experienced breast pathologist. The presence or absence of staining in peritumoral fibroblasts within 100 microns of the interface between tumor epithelium and stroma was performed by routine light microscopy. Identification of fibroblasts was primarily by morphologic criteria as the specificity of immunohistochemical markers for fibroblasts is poor. Therefore exclusion of cells that may be confused with fibroblasts was achieved by staining parallel sections with immunostains for endothelium (CD31) and inflammatory cells (CD68, CD45). The assessment of Tiam1 and osteopontin expression was determined by the presence or absence of stained fibroblasts and, to a lesser degree, by the intensity of the staining. Given variable fibroblast density among different breast cancer samples, the relative occurrence of stained fibroblasts within the 100 micron perimeter adjacent to pure ductal carcinoma in situ (DCIS) and pure invasive carcinoma was determined using a micrometer eyepiece. For both Tiam1 and OPN, greater than 50 % positive fibroblasts was counted as overexpression, and less than 10 % positive fibroblasts was counted as decreased expression. The average percentage of Tiam1-positive fibroblasts was 80.8 % for DCIS and 5.0 % in invasive carcinoma. The average percentage of OPN-positive fibroblasts was 6.9 % in DCIS and 77.5 % in invasive carcinoma.

### Approval for studies on animals and archived material from human subjects

All animal studies were conducted under approval of the Tufts University and Tufts Medical Center Institutional Animal Care and Use Committee in accordance with institutional and national regulatory standards (Tufts University and Tufts Medical Center Institutional Animal Care and Use Committee Protocol number B2013-51, PI Kuperwasser). Archived de-identified human breast cancer samples were obtained from the Tufts Cancer Center Repository, with waiver of need for informed consent granted by the Tufts Medical Center Institutional Review Board (IRB number 9138, certification of Exempt Status, PI Buchsbaum).

## Results

**Fig. 1:**
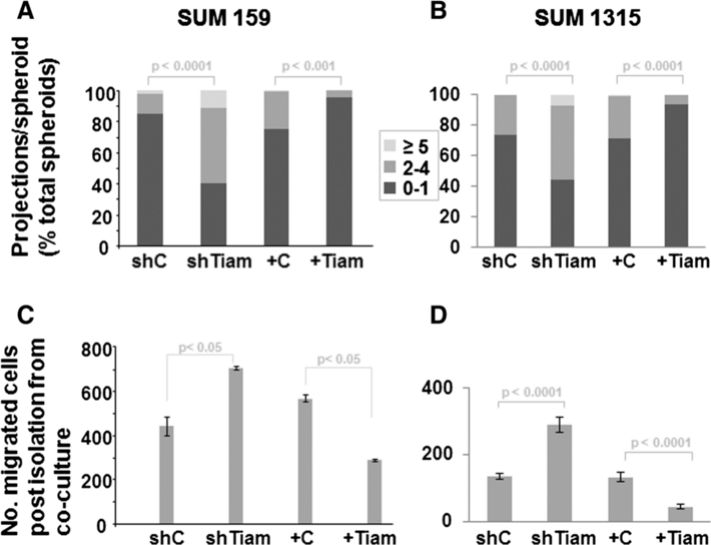
Effect of fibroblast Tiam1 expression on breast cancer cell invasion into co-culture matrix and migration of post-co-culture cells. **a** Number of projections/spheroid for SUM159 breast cancer cells and indicated mammary fibroblasts in 3D mixed cell spheroid co-culture. For fibroblasts: shC and shTiam refer to control silencing retroviral hairpin vector and Tiam1-silencing hairpin vector respectively; +C and + Tiam refer to pBabe control vector and pBabe-Tiam1 overexpression vector respectively. Results show number of spheroids with indicated number of projections as percent of total spheroids. At least 150 spheroids were counted for each condition. **b** Similar experiment as in *A* using SUM1315 breast cancer cells. At least 100 spheroids were counted for each condition. For *A* and *B*, results represent at least duplicate experiments. **c** Transwell migration of SUM159 after isolation from mixed cell spheroid co-cultures with indicated mammary fibroblasts. **d** Similar to *C* using SUM1315 breast cancer cells. For *C* and *D*, cell counts were averaged across nine high-power fields for biologic triplicates; results represent duplicate experiments. For fibroblasts in all experiments, *shC* = control silencing hairpin pSuperior vector; *shTiam1* = Tiam1 silencing; *+C* = pBabe overexpression vector; *+Tiam* = Tiam1 overexpression vector

To assess whether Tiam1 expression in mammary fibroblasts affects invasiveness of breast cancer cells in vitro, we adapted a 3D co-culture model of fibroblast and epithelial cell lines in extracellular matrix [35]. We have shown that co-culture of immortalized human mammary epithelial cells (HMECs) and immortalized human mammary fibroblasts (RMFs) in a Matrigel plug leads to assembly of 3D spheroid structures with fibroblasts clustering in the interior of the spheroid and epithelial cells arranged on the exterior of the spheroid [28]. Under conditions promoting invasion, the epithelial cells form multicellular outgrowths projecting out into the matrix. Invasiveness can be measured by counting the number of epithelial projections per spheroid [29]. We found that co-culture of several different breast cancer cell lines with RMFs leads to formation of similar spheroidal structures (not shown). In this study we tested the effect of manipulating fibroblast Tiam1 expression on the invasiveness of two aggressive breast cancer cell lines, SUM1315 and SUM159 [32] (Fig. 1). When cultured with control RMFs, most spheroids had 0–1 projection, but when cultured with RMFs with stable silencing of Tiam1 (shTiam1) there was a significant increase in the number of spheroids exhibiting increased numbers of projections. In contrast, spheroids with 0–1 projection increased and spheroids with higher numbers of projections decreased when cultured with RMFs overexpressing Tiam1. (Tiam1 expression in engineered RMFs is shown in Figure S1A in Additional file 2. All supplemental figures are included in the supplementary material [see Additional file 2]). This suggests that increased Tiam1 expression in mammary fibroblasts decreases invasiveness, and decreased Tiam1 expression increases invasiveness, in associated human breast cancer lines in a 3D environment.

In order to further assess the effects of mammary fibroblasts on associated human breast cancer cells, we derived a simple method for isolating enriched cancer cell populations after exposure in 3D co-culture [33], termed post-co-culture (PCC) cells. We have found that the effects of exposure to fibroblast in co-culture persist in PCC cancer cells for weeks after isolation, allowing for further study. We first tested the effect of exposure to Tiam1-modulated fibroblasts on migration of PCC breast cancer cells. Exposure to Tiam1-deficient fibroblasts increased migration across a perforated transwell, while exposure to Tiam1- overexpressing fibroblasts decreased migration (Fig. 1c, d).

### Tiam1 expression in fibroblasts modulates EMT in breast cancer cells isolated from 3D co-cultures

**Fig. 2:**
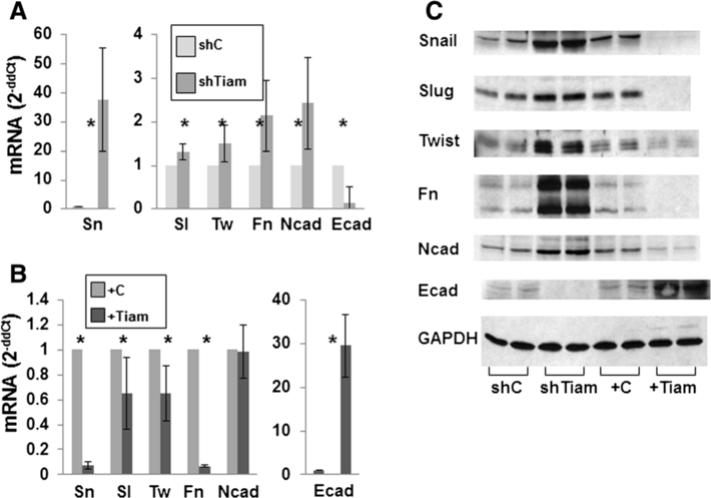
Effect of Tiam1 expression in fibroblasts on EMT in SUM1315 breast cancer cells isolated from 3D co-cultures. **a** Quantitative RT-PCR for mRNA of indicated proteins relative to GAPDH control from SUM1315 breast cancer cells isolated from 3D spheroid co-culture with control or Tiam1-deficient mammary fibroblasts. **b** Quantitative RT-PCR for mRNA of indicated proteins from SUM1315 breast cancer cells isolated from 3D spheroid co-culture with control or Tiam1-overexpressing mammary fibroblasts. For *A* and *B*, results represent mean +/- SD for duplicate experiments; ^*^Indicates *p* < 0.05 by *t* test. **c** Immunoblots from cell lysates of breast cancer cells derived as in *A* and *B*; biologic duplicates shown for each condition. *Sn* = Snail, *Sl* = Slug, *Tw* = Twist, *Fn* = Fibronectin, *Ncad* = N-cadherin, *Ecad* = E-cadherin

Since exposure to Tiam1-manipulated fibroblasts induced changes in invasion and migration in associated breast cancer cells, we then tested whether markers of epithelial-mesenchymal transition (EMT) in PCC-SUM1315 cells were also affected (Fig. 2). Exposure to Tiam1-deficient fibroblasts induced increases in the EMT profile, with a marked increase in Snail transcription (Fig. 2a). Smaller increases were seen in Slug, Twist, Fibronectin and N-cadherin, while E-cadherin transcription was decreased. Conversely, exposure to Tiam1-overexpressing fibroblasts decreased the EMT profile, with a marked decrease in Snail and Fibronectin, smaller decreases in Slug and Twist, and marked increase in E-cadherin transcription (Fig. 2b). Similar directional changes in EMT gene transcription profiles were found for SUM159 after exposure to Tiam1-deficient or Tiam1-overexpressing fibroblasts (Figure S2 in Additional file 2). Changes in gene transcription were paralleled by corresponding changes in protein expression (Fig. 2c). Thus exposure to Tiam1-altered fibroblasts in 3D induced changes in EMT consistent with induced changes in invasion and migration.

### Tiam1 expression in fibroblasts modulates markers of stem cell populations in breast cancer cells isolated from 3D co-cultures

**Fig. 3:**
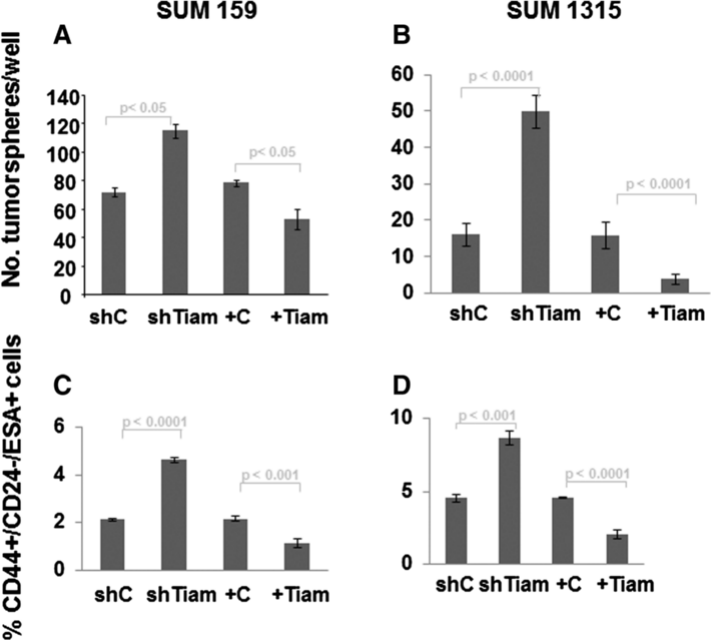
Effect of Tiam1 expression in fibroblasts on tumorsphere formation and CD44+/CD24-/ESA+ populations in breast cancer cells isolated from 3D co-cultures. **a** SUM159 breast cancer cells isolated from 3D co-cultures with indicated mammary fibroblasts were cultured at low density under ultra-low adherence conditions and tumorspheres were quantitated under light microscopy. **b** Similar experiment as in *A* using SUM1315 breast cancer cells. **c** Populations of SUM159 breast cancer cells were quantified by flow cytometry for expression of indicated cell surface markers using fluorophor-conjugated antibodies after isolation from 3D co-cultures with indicated fibroblasts. **d** Similar experiment as in *C* using SUM1315 cells. For *A-D*, results represent duplicate experiments, each with biologic triplicates

EMT has been associated with cancer stem cell populations [36]. We therefore investigated whether Tiam1 expression in mammary fibroblasts would affect cancer stem cell populations in associated breast cancer cells. We first determined whether the ability to nucleate a tumorsphere under low-adherence conditions was affected. Consistently, exposure to Tiam1-deficient fibroblasts induced increased numbers of tumorspheres in both SUM159 and SUM1315 PCC cells (Fig. 3a, b). Exposure to Tiam1-overexpressing fibroblasts led to decreased tumorsphere formation. While tumorsphere numbers were affected, tumorsphere size was not (Figure S1B in Additional file 2).

We also used flow cytometry to assess cancer stem cell populations in PCC cancer cells. The CD44^+^/CD24^-^/ESA^+^ subpopulation has been identified as being significantly enriched in the breast cancer stem cell population [32]. For both the SUM159 and SUM1315 lines, this population represented a small fraction of the cells after going through 3D co-culture with fibroblasts. However, exposure to Tiam1-deficient fibroblasts consistently led to approximate doubling of the CD44^+^/CD24^-^/ESA^+^ population, while exposure to Tiam1-overexpressing fibroblasts consistently led to a twofold decrease in the CD44^+^/CD24^-^/ESA^+^ population (Fig. 3c, d, and Figure S1C in Additional file 2). Thus the results of both tumorsphere assay and flow cytometry of cell surface markers suggested that Tiam1 expression in mammary fibroblasts modulates cancer stem cell-like populations in these breast cancer cell lines.

### Tiam1 expression in fibroblasts modulates breast cancer stem cell populations in vivo

The defining characteristics of cancer stem cells are the ability to initiate tumors in vivo and to maintain self-renewal. We therefore tested the ability of SUM1315 cells to initiate tumors in a mouse xenograft model after exposure to mammary fibroblasts in 3D co-culture. Xenograft implantations were established using PCC-SUM1315 breast cancer cells. Exposure to Tiam1-deficient fibroblasts significantly enhanced the ability of PCC-SUM1315 cells to initiate tumors (Table 1A). Conversely, exposure to Tiam1-overexpressing fibroblasts significantly impaired the ability of PCC-SUM1315 to initiate tumors.

**Table 1:**
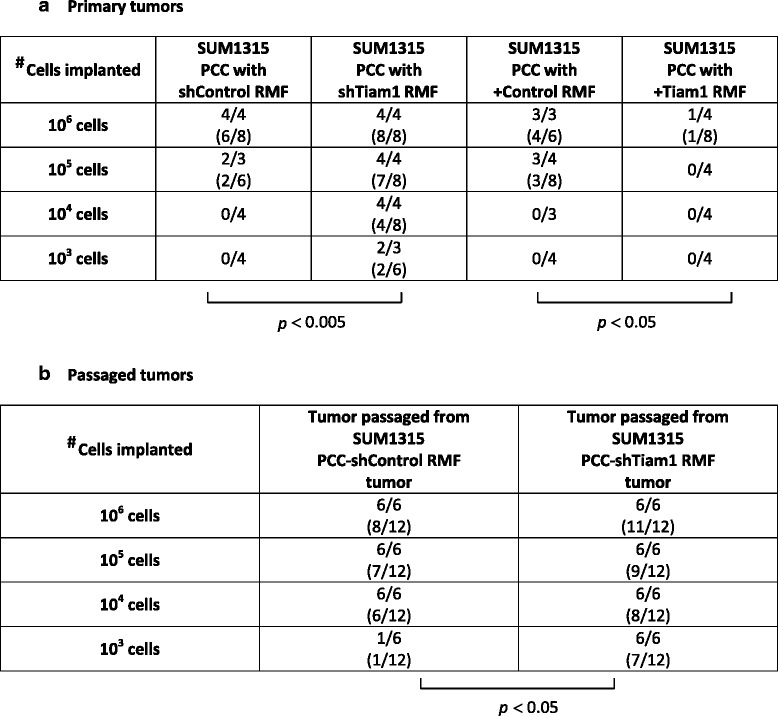
Effect of fibroblast Tiam1 on tumor-initiating ability of post-co-culture breast cancer cells in primary (A) and passaged (B) mouse xenografts

Results are shown as: ^#^tumor-bearing mice/total mice (^#^tumors/^#^xenograft implantations)

*P* values calculated by *t* test

*PCC* post-co-culture, *RMF* reduction mammary fibroblast

A. Primary xenografts were established with SUM1315 breast cancer cells under conditions of limiting dilution after isolation from 3D co-cultures with indicated mammary fibroblasts. B. Secondary passaged xenografts were established in serial dilution with single cell suspensions of primary tumors established in A, taken from the 10^6^ cell implantation and matched for size and weight

Self-renewal capability was tested by passage of primary tumors to secondary recipients. Three pairs of primary tumors arising from SUM1315 post-co-culture with control RMF or Tiam1-deficient RMF, matched for size, were passaged into secondary recipients. Co-culture with Tiam1-deficient fibroblasts led to primary tumors with significantly greater ability to give rise to secondary tumors at all implantation doses, particularly at low cell numbers (Table 1B). Thus varying Tiam1 expression in mammary fibroblasts modulated cancer stem cell properties in associated breast cancer cells.

### The effects of Tiam1-deficient fibroblasts on SUM1315 breast cancer cell invasion, migration, and cancer stem cell-like populations are dependent on fibroblast osteopontin

**Fig. 4:**
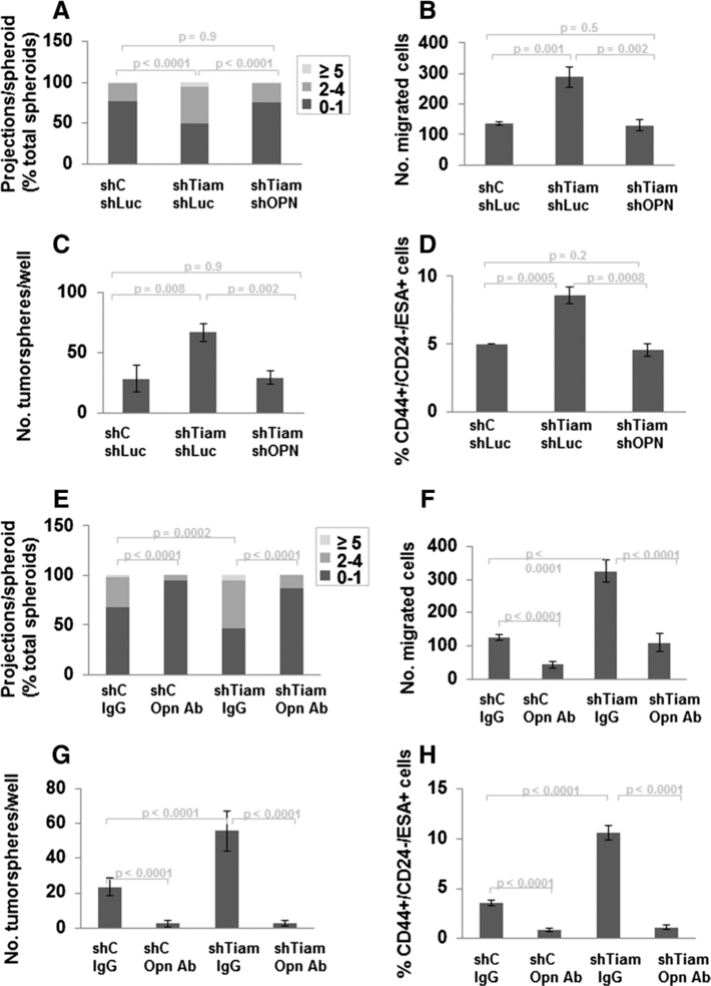
OPN inhibition prevents effects of Tiam1-deficient fibroblasts on breast cancer cell invasion, migration, and cancer stem cell-like populations. **a**-**d** Inhibition with OPN silencing. **a** Number of projections/spheroid for SUM1315 breast cancer cells and indicated mammary fibroblasts in 3D mixed cell spheroid co-culture. At least 130 spheroids were counted for each condition; results represent duplicate experiments. **b** Transwell migration of SUM1315 after isolation from mixed cell spheroid co-cultures with indicated mammary fibroblasts. Cell counts were averaged across nine high-power fields for biologic triplicates. **c** SUM1315 breast cancer cells isolated from 3D co-cultures with indicated mammary fibroblasts were cultured at low density under ultra-low adherence conditions and tumorspheres were quantitated under light microscopy. **d** Populations of SUM1315 breast cancer cells were quantified by flow cytometry for expression of indicated cell surface markers using fluorophor-conjugated antibodies after isolation from 3D co-cultures with indicated fibroblasts. For fibroblasts in *A-D*, *shC sLuc* = control silencing retroviral hairpin vector and luciferase silencing lentiviral hairpin vector; *shTiam shLuc* = Tiam1 and luciferase silencing hairpins; *shTiam shOPN* = Tiam1 and OPN silencing hairpins. **e**–**h** Inhibition with OPN antibody. Results of mixed cell co-cultures established as in Fig. 1a with SUM1315 breast cancer cells and either control (*shC*) or Tiam1-deficient (*shTiam*) fibroblasts, incorporating IgG or OPN antibody as indicated. **e** Number of projections/spheroid. At least 180 spheroids were counted for each condition; results represent duplicate experiments. **f** Transwell migration of SUM1315 after isolation from mixed cell spheroid co-cultures with indicated mammary fibroblasts. Cell counts were averaged across nine high-power fields. **g** SUM1315 breast cancer cells isolated from 3D co-cultures with indicated mammary fibroblasts were cultured at low density under ultra-low adherence conditions and tumorspheres were quantitated under light microscopy. **h** Populations of SUM1315 breast cancer cells were quantified by flow cytometry for expression of indicated cell surface markers using fluorophor-conjugated antibodies after isolation from 3D co-cultures with indicated fibroblasts. For b, c, d, f, g, and h results represent duplicate experiments, each with biologic triplicates

We have found that stress-induced senescence induces decreased Tiam1 expression in mammary fibroblasts and that Tiam1 decrease in fibroblasts is associated with increased expression and secretion of fibroblast OPN [29]. We therefore asked whether the effects of Tiam1-deficient fibroblasts on breast cancer cell behavior were dependent on fibroblast OPN. We first tested the effect of silencing OPN expression in mammary fibroblasts (Fig. 4). (OPN transcription in engineered RMFs is shown in Figure S1D in Additional file 2.) Co-culture of SUM1315 with fibroblasts deficient in both Tiam1 and OPN completely abrogated the increased invasiveness induced by co-culture with Tiam1-deficient fibroblasts (Fig. 4a). Concordantly, PCC SUM1315 isolated from co-cultures with fibroblasts deficient in both Tiam1 and OPN also displayed decreased migration, tumorsphere formation, and CD44^+^/CD24^-^/ESA^+^ populations (Fig. 4b–d), compared with SUM1315 isolated from co-cultures with Tiam1-deficient fibroblasts.

We also tested the ability of an OPN antibody to block the effects of Tiam1-deficient fibroblasts in these assays. Incorporation of an OPN antibody into the matrix and medium of the co-cultures led to a significant decrease in numbers of invading projections, independent of the Tiam1 expression of the co-cultured RMFs (Fig. 4e). As OPN is secreted by multiple cell types, including cancer cells, we investigated whether the incorporated OPN antibody was primarily affecting fibroblast-derived OPN or SUM1315-derived OPN. We found that OPN transcription is significantly less in SUM1315 cells than in RMF cells (Figure S3A in Additional file 2). We then tested the effect of OPN inhibition on migration of SUM1315 cells using differential incorporation of OPN antibody against SUM1315 cells or RMF cells (Figure S3B in Additional file 2). Overall SUM1315 migration was increased toward Tiam1-deficient fibroblasts (*darker bars*). Incorporating OPN antibody against SUM1315 cells (in the SUM1315 culture and in the top well) had no effect on SUM1315 migration. Incorporating OPN antibody against RMF cells (in the bottom well) significantly blocked SUM1315 migration. These results suggest that the major effect of the OPN antibody in these co-cultures is against fibroblast-derived OPN, and are consistent with our findings that OPN silencing in RMF is sufficient to block all of the effects of RMF Tiam1 silencing on associated SUM1315 cancer cells (Fig. 4a–d). Correspondingly, SUM1315 isolated from co-cultures incorporating OPN antibody displayed decreased migration, tumorsphere formation, and CD44^+^/CD24^-^/ESA^+^ populations (Fig. 4f–h). Taken together, these findings suggest that the effects of fibroblast Tiam1 deficiency on SUM1315 breast cancer cell behavior are dependent on fibroblast OPN.

### OPN inhibition during co-culture of SUM1315 breast cancer cells and mammary fibroblasts prevents development of lung metastases in mice

Tiam1-deficient fibroblasts promote metastasis in a mouse xenograft co-implantation model of human breast cancer [28]. Given the results above of OPN inhibition by gene silencing or antibody in vitro, we tested the effects of a chemical inhibitor of OPN in blocking the effects of Tiam1-deficient RMF in an accelerated in vivo model of breast cancer metastasis. Agelastatin A (AgelA) is a brominated oroidin alkaloid originally of marine origin, and synthetic AgelA inhibits OPN transcription and expression [37]. One of our laboratories has developed a concise enantioselective solution for the total chemical synthesis of AgelA [34, 38]. We therefore tested the ability of synthesized AgelA to block the effects of Tiam1-deficient fibroblasts on SUM1315 breast cancer cells in our systems.

We first tested the cytotoxicity of AgelA in a proliferation assay of RMFs in 2D culture (Figure S4a in Additional file 2). Twenty-five, 50, and 100 nM AgelA had no statistically significant effect on cell proliferation. Notable decreases in proliferation were seen at concentrations higher than 100 nM, consistent with prior reports of cytotoxicity [37]. Similarly, treatment with AgelA 75 nM had no direct effect on SUM1315 proliferation (Figure S4b in Additional file 2).

We then tested whether AgelA at non-cytotoxic concentrations could block the induction of OPN expression by vitamin D, a known inducer of OPN transcription [39]. Vitamin D induced an almost twofold increase in OPN transcription, which was blocked by concurrent treatment with increasing doses of AgelA (Figure S4c in Additional file 2). Notably, 50–100 nM of AgelA prevented any significant vitamin D-induced increase in OPN transcription. AgelA treatment had no effect on Tiam1 expression (not shown).

We next tested the effect of incorporating AgelA into our co-cultures and post-co-culture assays. Increasing doses of AgelA in the co-culture matrix and medium led to decreasing SUM1315 invasion into 3D matrix (Figure S5a in Additional file 2). Notably, 100 nM AgelA almost completely abolished the increased invasiveness induced by Tiam1-deficient fibroblasts, compared with vehicle alone. In the biologic assays of PCC-SUM1315, we also found corresponding decreases in migration, tumorsphere formation, and CD44^+^/CD24^-^/ESA^+^ populations (Figures S5b, S6a-b in Additional file 2).

We also assessed whether AgelA treatment could have direct functional effects on breast cancer cells by assaying SUM1315 cells grown alone under standard 2D tissue culture conditions. As noted above (Figure S3 in Additional file 2), there is very little OPN transcription in SUM1315 cells compared with RMF cells, and this was not significantly affected by AgelA treatment (not shown). Consistent with this, we found that treatment with AgelA 75 nM had no effect on migration, tumorsphere formation, or CD44^+^/CD24^-^/ESA^+^ populations of SUM1315 in 2D culture (not shown). The effects of AgelA on the behavior of SUM1315 depended on exposure to RMF in 3D mixed cell co-cultures.

In comparative experiments, we also investigated whether treatment with chemotherapy would have similar effects as AgelA on invasion, migration, and cancer stem cell population. We first determined maximal tolerated concentrations for doxorubicin, docetaxel, and cyclophosphamide that did not affect cell growth, arriving at final concentrations of doxorubicin 0.8 nM, docetaxel 0.95 nM, and cyclophosphamide 1000 nM. We then established co-cultures with SUM1315 and either control or Tiam1-deficient fibroblasts as detailed above, incorporating each chemotherapeutic agent or control media into the co-cultures. Co-culture with Tiam1-deficient fibroblasts induced increased SUM1315 invasion into the 3D matrix (Figure S7a in Additional file 2). However, no chemotherapeutic agent prevented increased invasiveness, in contrast to AgelA. Similarly, no chemotherapeutic agent prevented increased PCC SUM1315 migration, tumorsphere formation, or CD44+/CD24-/ESA+ populations induced by exposure to Tiam1-deficient fibroblasts (Figures S7b, S8a-b in Additional file 2).

**Fig. 5:**
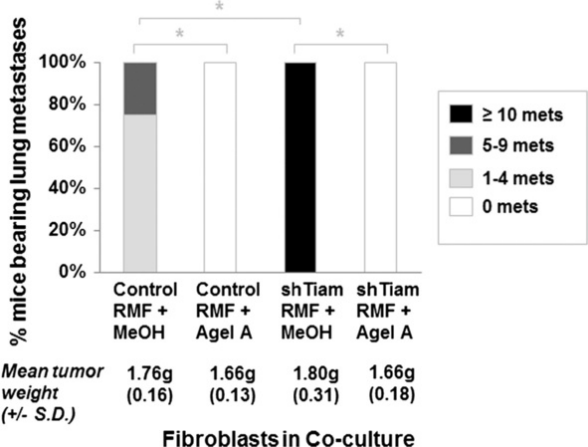
Effect of Agelastatin A treatment of co-cultured breast cancer cells on development of lung metastases in a mouse xenograft model. Percent of xenograft-bearing mice with indicated degree of detectable lung metastases after implantation with SUM1315 breast cancer cells isolated from co-cultures with indicated fibroblasts and incorporation of AgelA or MeOH at equivalent dilution in the cultures. Mean tumor weights +/- standard deviation (SD) are indicated for each cohort. ^*^ = *p* < 0.5 by Fisher’s exact test

We therefore tested the effects of AgelA in an accelerated model of metastasis in a mouse xenograft model (Fig. 5). We established co-cultures with SUM1315 and control or Tiam1-deficient RMF, incorporating either 75nM AgelA or vehicle (methanol [MeOH]). PCC-SUM1315 from each co-culture were implanted into the fourth mammary fat pads of immune-deficient mice. All mice developed palpable primary tumors within 5 weeks, with similar tumor growth. In contrast, there was a clear difference in metastatic burden in the lungs, as assessed by immunohistochemistry (IHC). Implantation of PCC-SUM1315 exposed to control RMF and vehicle led to a low level of lung metastasis. Implantation of PCC-SUM1315 exposed to Tiam1-deficient RMF and vehicle led to a high level of lung metastasis (three mice with foci that were too numerous to count). In contrast, incorporation of AgelA into the co-cultures completely abolished the lung metastases, even though primary tumor growth was unaffected.

### In human breast cancer-associated fibroblasts, Tiam1 expression varies inversely with cancer invasiveness, while OPN expression varies directly with cancer invasiveness

**Fig. 6:**
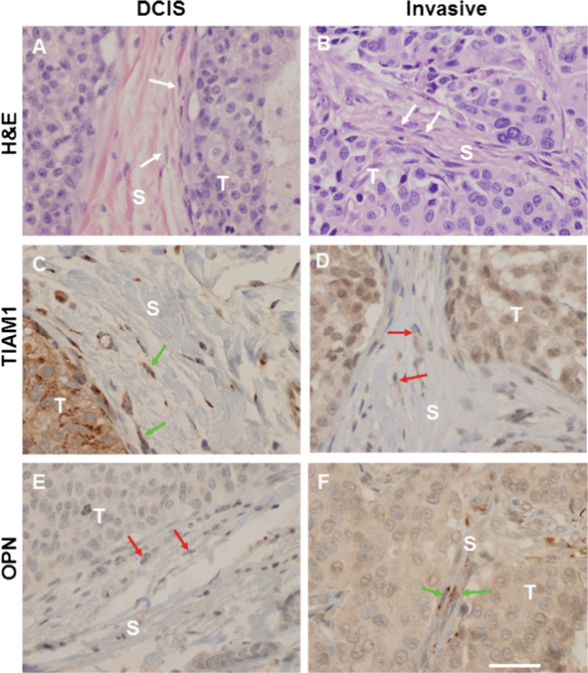
Expression patterns of Tiam1 and OPN in breast cancer-associated fibroblasts, correlated with invasiveness. Representative sections of non-invasive (DCIS) and invasive breast cancers showing tumor cells (*T*) with interspersed stroma (*S*), stained with hematoxylin and eosin (*H&E*), Tiam1 antibody, or OPN antibody as indicated. **a** and **b** *White arrows* indicate fibroblast nuclei in stroma adjacent to tumor cells. **c** and **f** *Green arrows* indicate fibroblasts with detectable overexpression of indicated protein. **d** and **e** *Red arrows* indicate fibroblasts without overexpression of indicated protein. All images taken at 40× magnification, scale bar indicates 100 μM

Finally, to evaluate the possible role of the fibroblast Tiam1-OPN pathway in human cancer, we investigated the expression of Tiam1 and OPN in fibroblasts associated with human breast cancers using IHC (Fig. 6). In a pilot study we found Tiam1 protein expression to be readily detectable in cancer-associated fibroblasts in 15 of 17 cases of DCIS (ductal carcinoma in situ), but decreased in cancer-associated fibroblasts in 19 cases of invasive cancers (*p* < 0.001). In an expanded study, 36 independent surgical specimens from 26 individual cases of breast cancer (invasive and/or in situ ductal carcinomas) had tissue available for IHC for Tiam1 and/or OPN expression. In 20 areas of invasive ductal carcinoma, cancer-associated fibroblasts showed increased cytoplasmic OPN expression and decreased Tiam1 expression. In 19 areas of DCIS, cancer-associated fibroblasts showed decreased expression of OPN and increased cytoplasmic expression of Tiam1. Clinical characteristics of the tumors are shown in Table S1 (see Additional file 3). These findings were independent of tumor grade, lymph node status, estrogen receptor (ER) and progesterone receptor (PR) expression. Of note, while human epithelial receptor 2 (HER2) expression ranged from 0 to 3+ by IHC staining, there were no cases of HER2 amplification by fluorescence in situ hybridization (FISH) in this cohort. Expression of Tiam1 and OPN in the tumors themselves did not correlate with invasiveness. These results show that expression of Tiam1 and OPN in breast cancer-associated fibroblasts are inversely related to each other and correlate with tumor invasiveness.

## Discussion

We have previously shown that silencing the Rac exchange factor Tiam1 in tumor-associated fibroblasts induces increased invasion and metastasis in epithelial and cancer cells, using different tissue models with a range of technical complexity and biologically relevant tissue context. Here we investigate the underlying mechanism in a breast cancer-oriented system, using a novel method for isolating human breast cancer cells out of 3D spheroid co-cultures containing mammary fibroblasts. Co-culture of human breast cancer cells with Tiam1-deficient fibroblasts induces increased cancer cell invasion into matrix, while co-culture with Tiam1-overexpressing fibroblasts induces decreased invasion. Strikingly, breast cancer cells isolated from 3D culture with Tiam1-deficient fibroblasts exhibit persistent increases in migration, EMT, and cancer stem cell characteristics. In contrast, breast cancer cells exposed to Tiam1-overexpressing fibroblasts and then isolated from 3D co-cultures persistently display the opposite phenotypes. The observed changes in the SUM1315 breast cancer cells in our system are dependent on fibroblast-produced OPN, as OPN silencing or antibody-mediated inhibition abrogates the increased invasion, migration, and cancer stem cell properties induced by Tiam1-deficient fibroblasts. Treatment of co-cultures by chemotherapeutic agents does not prevent effects induced by Tiam1-deficient fibroblasts. In contrast, treatment of co-cultures by the OPN inhibitor Agelastatin A reverses all effects induced by Tiam1-deficient fibroblasts, and notably blocks subsequent development of lung metastases in the mouse xenograft model. Finally, in human breast cancer samples Tiam1 expression is significantly decreased in fibroblasts associated with invasive human breast cancers compared to fibroblasts associated with ductal carcinoma in situ (DCIS). Conversely, OPN expression is significantly increased in fibroblasts associated with invasive breast cancers compared to fibroblasts associated with DCIS, suggesting the relevance of fibroblast Tiam1 and OPN expression to the behavior of human breast cancers.

Our results suggest several corollary findings. The effects of the fibroblasts on the co-cultured cancer cells are long-lived rather than transient and do not require ongoing exposure to the fibroblasts, since changes in post-co-culture cells persist for many weeks in vitro and the results in vivo shown in Table 1 represent effects on cancer stem cell populations manifesting several months after isolation from co-culture with fibroblasts. This suggests that the mechanism for these effects is more likely to depend on changes in gene expression (such as epigenetic alterations), rather than changes in signaling pathways per se. Active investigation of the underlying mechanism is underway. In addition, the fact that the OPN inhibitor Agelastatin A blocked SUM1315 lung metastasis but not primary tumorigenesis in mouse xenografts established with isolated post-co-culture cancer cells is consistent with our earlier report that co-implantation of breast cancer cells with Tiam1-deficient fibroblasts affected metastasis but not primary tumorigenesis [28]. This strongly suggests a role for fibroblast Tiam1-OPN pathway in regulating breast cancer metastasis specifically. Finally, the model of orthotopic xenograft implantation of cancer cells isolated from fibroblast co-culture leads to established metastatic disease more rapidly (less than 8 weeks) than orthotopic mixed cell (fibroblast-cancer cell) implantation (20 weeks), which may allow more practicable future study of factors affecting tumor metastasis in vivo.

How Tiam1 expression might regulate OPN expression is not clear. Tiam1 participates in multiple signaling pathways with diverse Rac effector proteins, many of which are mediated through Tiam1 interaction with diverse scaffolding proteins responding to cell-context specific signals [40]. Whether the link to OPN regulation is through one of the known Tiam1 pathways or an undiscovered pathway has yet to be determined. Regulation of the promoter-driven expression of OPN is quite complex, with multiple contributing promoter polymorphisms and cis-regulatory elements (reviewed in [31, 41]). Of note, Tiam1 directly interacts with activated Ras through a Ras-binding domain and has also been identified as a target of Wnt signaling [14], suggesting two potential areas of overlap with known OPN regulators for further study.

In addition, the OPN gene (also known as SPP1) has alternative start sites and the OPN protein undergoes extensive post-translational modification, including phosphorylation, glycosylation and proteolytic cleavage, leading to complex effects. Various forms of OPN are secreted from multiple cell types, both malignant and non-malignant. Isoform and post-translational modification changes are cell-type specific, but not necessarily cancer-specific [42]. Most reports on stromal OPN center on macrophage-derived OPN. There are limited reports of the effect of fibroblast-derived OPN in disease. Senescent fibroblasts, like cancer-associated fibroblasts, stimulate neoplastic and preneoplastic cell growth [43–45]. In preneoplastic human keratinocytes, this depended on keratinocyte MAPK activation due to dermal fibroblast OPN secretion [45, 46]. A microdialysis-proteomics study of the mammary tumor microenvironment in PyVmT transgenic mice identified OPN in tumor-associated fibroblasts as well as tumor cells, and recombinant OPN stimulated proliferation of PyVmT carcinoma cells in vitro [47]. Which form of OPN might be operating in tumor-associated fibroblasts, and how effects of fibroblast-derived OPN differ from tumor-derived OPN, are not yet fully understood.

In experimental systems, cancer-associated fibroblasts (CAF) are heterogeneous, suggesting several subsets within the broad umbrella of the CAF label (reviewed in [48, 49]). Recent reports suggest that tumor-derived OPN induces expression of CAF-associated markers in mesenchymal stromal cells through upregulation of transforming growth factor beta 1 (TGF-β1) [50, 51]. Another study showed that tumor-derived OPN has a key role in reprogramming normal mammary fibroblasts to an inflammatory CAF-like phenotype, and found evidence of autocrine OPN effects within the induced CAFs themselves [52]. A study of B16 mouse melanoma engineered to express platelet-derived growth factor (PDGF) showed that tumor-derived PDGF induces CAF recruitment and OPN expression in a subset of recruited CAFs [53]. A search of the Oncomine database (www.oncomine.org) identifies widely variable changes in SPP1 expression across a wide range of tumor cell-related data sets, but the three datasets focusing specifically on breast cancer stroma demonstrate changes supporting our hypothesis. In the Karnoub breast data set, SPP1 expression was 16-fold higher in the stroma of invasive ductal breast cancers (*n* = 7) compared with normal breast stroma (*n* = 15), with *p* = 2.875E-5 [54]. In the Finak breast data set, invasive breast cancers with recurrence over 3 years (*n* = 59) showed fourfold increase in SPP1 compared with normal (*n* = 6), with *p* = 1.06E-4 [55]. Conversely, in the Ma data set there was no significant change in SPP1 in DCIS-associated stroma compared with normal [56]. Of note, none of these data sets found significant changes in Tiam1 expression, which may indicate a post-translational mechanism for Tiam1 downregulation in the tumor microenvironment [18, 20].

While there have been striking advances in the management of early stage breast cancers, the same is not true for metastatic breast cancer, and the death rate from metastatic breast cancer has not changed significantly over decades. At present, treatments for metastatic breast cancer involve therapies based on the traditional model of reducing tumor cell bulk, either by inducing general cytotoxic effects on the cells or targeting specific pathways in cancer cells that lead to bulk cell death. Current understanding in cancer biology includes the concept of cancer stem cell populations, which often comprise a very small percent of the primary tumor bulk but a large percent of the cells giving rise to relapse and metastasis. Cancer stem cell populations are relatively resistant to traditional therapeutic approaches and may be a key mechanism underlying dormancy in breast cancers, leading to late metastatic relapses years after apparent successful treatment of primary tumors. There are early suggestions that in some systems, notably glioma and colon cancer, OPN may promote stem-cell like changes through triggering expression and signaling of its cell surface receptor CD44 [57, 58]. These are consistent with our findings that inhibiting OPN in tumor-associated fibroblasts diminishes cancer stem cell populations and blocks metastatic potential in an aggressive breast cancer line.

Finally, while our experimental results were derived largely with aggressive breast cancer cell lines that do not express estrogen or progesterone receptors, we found that the expression pattern of Tiam1 and OPN in human breast cancer-associated fibroblasts was correlated with invasiveness rather than hormone receptor expression. Most of the human breast cancer samples we studied had at least some expression of ER and/or PR, consistent with the pattern of breast cancers seen in clinical practice. Study of larger sample size will be required to fully explore whether the Tiam1-OPN expression patterns we have observed are limited to the stroma around particular breast cancer subsets, or whether this is a general characteristic across multiple breast cancer subsets. Furthermore, fibroblasts associated with human breast cancers are likely comprised of heterogeneous cell populations, like experimentally derived CAFs and breast cancer cells themselves. It may be that the fibroblast Tiam1-OPN pathway is most relevant in vivo in specific fibroblast subsets. Our findings using immortalized mammary fibroblasts and specific breast cancer cell lines lay the foundation for studying these questions in broader contexts.

## Conclusions

In summary, our results suggest that regulated Tiam1 expression in breast cancer-associated fibroblasts modulates breast cancer invasion, EMT, cancer stem cell populations, and metastasis through regulation of fibroblast OPN secretion. At least some human breast cancers demonstrate commensurate changes in fibroblast Tiam1 and OPN expression correlating with invasiveness. This suggests that the fibroblast Tiam1-OPN pathway may offer both novel therapeutic targets as well as novel biomarkers in the treatment of metastatic breast cancer.

## Additional files

Additional file 1:
**Additional details for experimental methods as indicated in the main Methods section, including derivation of sublines, co-cultures, transwell migration assay, tumorsphere assay, flow cytometry, RT-PCR, and tumor passage.** (DOCX 31 kb) Additional file 2:
**Supplemental figures S1-S8 and supplemental figure legends.** (PDF 467 kb) Additional file 3:
**Supplemental Table S1.** (DOCX 18 kb)

## Abbreviations

3D: Three-dimensional
AgelA: Agelastatin A
CAF: Cancer-associated fibroblasts
DCIS: Ductal carcinoma in situ
EMT: Epithelial-mesenchymal transition
ER: Estrogen receptor
GFP: Green fluorescent protein
HER2: Human epithelial receptor 2
IgG: Immunoglobulin G
IHC: Immunohistochemistry
MeOH: Methanol
OPN: Osteopontin
PCC: Post-co-culture
PDGF: Platelet-derived growth factor
PR: Progesterone receptor
RMF: Reduction mammary fibroblast
RNA: Ribonucleic acid
RT-PCR: Reverse transcriptase polymerase chain reaction
