# Supplementary material for: The fibroblast Tiam1-osteopontin pathway modulates breast cancer invasion and metastasis
Source: Breast Cancer Res. 2016 Jan 28;18:14. doi: 10.1186/s13058-016-0674-8 (PMC4730665; doi:10.1186/s13058-016-0674-8)
Supplement: Additional file 1: — Additional details for experimental methods as indicated in the main Methods section, including derivation of sublines, co-cultures, transwell migration assay, tumorsphere assay, flow cytometry, RT-PCR, and tumor passage. (DOCX 31 kb) [file 13058_2016_674_MOESM1_ESM.docx]

**Supplemental File: Materials & Methods**

**Cell culture**

All reduction mammary fibroblast lines (RMFs) were grown in Dulbecco’s modified Eagle’s medium (DMEM, Mediatech, Inc.) supplemented with 10% bovine calf serum. The human breast cancer line, SUM1315, was grown in Ham’s F12 nutrient mixture (Gibco) with 5% fetal bovine serum (Gibco), 10 ng/mL EGF, and 5 ug/mL insulin. The human breast cancer line, SUM159, was grown in DME/F12 medium (Gibco) with 5% fetal bovine serum, 5 ug/mL insulin, and 0.5 ug/mL hydrocortisone. All culture media contained 100 Units/mL penicillin and 100 ug/uL streptomycin and 0.1% fungizone.

The cell lines used here have been described previously, as follows:

the Tiam1-deficient shTiam-RMF and the retroviral hairpin control RMF (C-RMF) lines [[25](#_ENREF_25)];

the Tiam1-over-expressing (+Tiam) RMF line, the pBabe vector control RMF line, the Tiam1 and OPN-deficient shTiam-OPN RMF line and the double retroviral-lentiviral control line [[26](#_ENREF_26)];

the human breast cancer cell lines SUM1315 and SUM159 [[29](#_ENREF_29)].

**Spheroid co-culture**

Co-cultures were established as previously described [[30](#_ENREF_30)]. Images were obtained on a Diaphot TMD Nikon Inverted Tissue Culture Microscope using a Spot RT-SE camera and SPOT Software Version 4.1 (Diagnostic Instruments Inc., Sterling Heights, MI, USA). Where indicated, the following agents were incorporated into the Matrigel and culture medium: 667 ng/mL anti-OPN antibody (Santa Cruz Biotechnology); 667 ng/mL isotypic IgG control antibody; 75 nM Agelastatin A; 750 uM cyclophosphamide; 1 nM docetaxel; 8 nM doxorubicin. AgelA was synthesized as previously described [[33](#_ENREF_33)]. Stock solutions (1mM) were stored in methanol at -20^o^C, and diluted in culture medium to indicated final concentrations.

**Isolation of cells from spheroid co-culture**

Cells were isolated from 3D co-cultures and cancer cells were separated from fibroblasts as described previously [[30](#_ENREF_30)]. Flow cytometry was used to demonstrate purity of cells isolated post-co-culture to greater than 99% cancer cells using fluorescent markers (RMFs with GFP, cancer cells with or without pCherry).

**Growth curves**

RMF or SUM1315 cells were seeded at 3 x10^5^ cells per 100-cm culture dish in appropriate culture medium in the absence or presence of AgelA at final indicated concentrations, and passed every two days. At each passage, 3 x10^5^ cells were seeded into a new dish. AgelA was continued in the cultures at indicated concentrations throughout the experiment. Total number of cells in each dish was calculated as the number of cells in the dish at each time point corrected for the fractional passages.

**Immunoblotting**

Immunoblotting was performed as previously described [[25](#_ENREF_25)]. Blots were developed using ExtraChemiLuminscence (Amersham) and exposed to autoradiography. Images were scanned using HP ScanJet 4570C scanner with HP Scan Pro program (V6.1.3) and digitized using the Image J 1.410 software for quantification. Antibodies were obtained from the following sources: Tiam, GAPDH, Fibronectin, Twist (Santa Cruz); Snail, Slug, Ecadherin, Ncadherin (Cell Signaling).

**Transwell migration assay**

Transwell migration was assessed as previously described [[26](#_ENREF_26)].

**Tumorsphere Assays**

Cancer cells were trypsinized and cell clumps were broken up by gentle pipetting several times. After low-speed centrifugation (1200 rpm), cell pellets were resuspended in corresponding culture medium, and passed through a 40-μm filter to obtain single cell suspensions. Five thousand cells in 4 mL culture medium were plated per well in ultra-low attachment 6-well plates (Costar). Tumorspheres were counted within 10 to 14 days in culture and sphere images were obtained on a Diaphot TMD Nikon Inverted Tissue Culture Microscope using a Spot RT-SE camera and SPOT Software Version 4.1 (Diagnostic Instruments Inc., Sterling Heights, MI, USA).

**Flow Cytometry**

Aliquots of 0.5X10^6^ cells in 100 μL of FACS buffer (phosphate-buffered saline with 2% BSA) were stained with EpCAM (ESA)-FITC (clone VU-ID9, AbD Serotec, 1:10), CD24-PE (clone ML5, BD Pharmingen, 1:400), and CD44-APC (clone G44-26, BD Pharmingen, 1:50) antibodies in the dark at room temperature for 30 min. At the end of incubation, 900 μL of FACS buffer were added to the cell/antibody mixture, and cells were then analyzed using Beckman Coulter CyAn_ADP_ with Summit 4.3 program. To set background gating, other cell aliquots were stained with isotype control antibodies conjugated with corresponding fluorophor (APC mouse IgG2b k, BD Biosciences; PE mouse IgG2a k, BD Biosciences; FITC mouse IgG, Becton Dickinson Immunocytometry systems) so that the nonspecific CD44+/CD24-/ESA+ staining represented less than 0.5% of the cell populaton. Cells were first gated using APC and PE to identify the CD44+/CD24- population, followed by secondary gating on FITC to identify the CD44+/CD24-/ESA+ cells.

**Real time PCR**

Isolation of RNA and quantitative RT-PCR were performed as previously described [[26](#_ENREF_26)], using the following primer pairs:

Snail, 5’-AATCGGAAGCCTAACTACAGCG-3’ (sense), 5’-GTCCCAGATGAGCATTGGCA-3’ (antisense);

Slug, 5’-AAGCATTTCAACGCCTCCAAA-3’ (sense), 5’-AGGATCTCTGGTTGTGGTATGAC-3’ (antisense);

Twist, 5’-GTCCGCAGTCTTACGAGGAG-3’ (sense), 5’-GCTTGAGGGTCTGAATCTTGCT-3’ (antisense);

Fibronectin, 5’-CAGTGGGAGACCTCGAGAAG-3’ (sense), 5’-TCCCTCGGAACATCAGAAAC-3’ (antisense);

NCadherin, 5’-TCCTACTGGACGGTTCGCCA-3’ (sense), 5’-TTGCAGTTGACTGAGGCGGG-3’ (antisense);

ECadherin, 5’-CCCACCACGTACAAGGGTC-3’ (sense), 5’-ATGCCATCGTTGTTCACTGGA-3’ (antisense);

GAPDH, 5’-CTGCACCACCAACTGCTTAG-3’ (sense), 5’-TTCAGCTCAGGGATGACCTT-3’ (antisense);

Tiam1, 5’- AAGACGTACTCAGGCCATGTCC-3’ (sense), 5’-GACCCAAATGTCGCAGTCAG-3’ (antisense);

OPN, 5’-GCCATACCAGTTAAACAGGC -3’ (sense), 5’- GACCTCAGAAGATGCACTAT -3’ (antisense).

**Human breast cancer xenograft models**

All animal studies were conducted under approval of the Tufts University and Tufts Medical Center Institutional Animal Care and Use Committee in accordance with institutional and national regulatory standards.

*Primary tumors:*

Eight-week-old female non-obese diabetic/severe combined immunodeficient (NOD/SCID) mice were purchased from Jackson Laboratory (Bar Harbor, ME, USA). SUM1315-GFP/luc cells (2.5x10^5^) were established in spheroid co-cultures with either control, Tiam1-deficient, or Tiam1-overexpressing fibroblasts as indicated for 2 weeks. After recovery from co-culture, cells were cultured in tissue culture dishes in SUM1315 medium for 10-12 days. After trypsinization, PCC non-fluorescing SUM1315 cells were further purified from GFP-expressing RMFs by flow cytometry, suspended in a mixture of Matrigel and SUM1315 culture medium (1:3 ratio), and injected into fourth inguinal mammary glands in a 35 μL volume. Tumor growth was monitored weekly by manual measurement using electronic digital caliper (Control Company, TX, USA).

*Passaged tumors*:

Isolation of single tumor cells

Under sterile conditions, excised primary tumors were minced in 1 mL PBS in 100-mm culture dishes until no chunks were visible. After addition of 9 mL PBS to the dish, the contents were transferred to 15 mL conical tubes and centrifuged at 1200 RPM for 5 min. Pellets were resuspended in epithelial cell medium (DMEM/Ham’s F12 (1:1) medium supplemented with 5% Bovine Calf Serum, 10 ng/mL insulin, 10 ug/mL EGF, 10 ug/mL hydrocortisone, 100 Units penicillin, 100 ug/uL of streptomycin, and 0.1% fungizone) containing 1.5 mg/mL collagenase (Roche) and 125 U/mL hyaluronidase (Sigma). Minced tumors were incubated at 37^o^C with rotation for 3 hours, centrifuged as above, washed 3 times with 5% calf serum in PBS, and pellets were resuspended in 10 mL SUM1315 culture medium. Resuspended pellets were passed 8-10 times through an 18G needle attached to a 10 mL syringe, centrifuged as above, washed once with PBS, and finally resuspended in 2 mL 0.05% Trypsin/0.53 mM EDTA. Suspensions were then incubated at 37^o^C for 10 min, and cell aggregates were broken up by pipetting every two to three minutes. Eight ml of cell culture medium were then added along with 62.5 μg/mL DNAse solution (Sigma) at a final concentration of 50μg/mL. Cell suspensions were filtered through 40 μm cell strainer (BD Biosciences), centrifuged as above, resuspended in 10 mL RMF culture medium, and cultured for 1 hour at 37^o^C in order to allow stromal cells to adhere. Non-adherent tumor cells were then collected without trypsinization, counted, and suspended in a mixture of Matrigel and SUM1315 culture medium (1:3) for injection as described above.

*Tumor and organ harvesting*

Mice were sacrificed when appearing ill or when total tumor bulk reached 2 cm. Tumors and organs were harvested according to established protocols as described previously [[25](#_ENREF_25)].

**Immunohistochemical staining of human tumor samples**

Human breast cancer samples were obtained from the Tufts Cancer Center Repository under approval of the Tufts Medical Center Institutional Review Board. Immunohistochemistry was performed using 5 micron sections of formalin fixed human tissue. Samples were stained on the Ventana Benchmark XT (Ventana Medical Systems, Inc. Tucson, Arizona). Tiam-1 staining was done using a mild retrieval protocol (CC1 for 30 minutes) with Tiam-1 antibody (Santa Cruz sc-872) at dilution of 1:200 for 32 minutes. Osteopontin staining was done using the standard protocol for retrieval (CC1 for 60 minutes) with Osteopontin antibody (Abcam ab33046) at a dilution of 1:1000 for 16 minutes.
