# Supplementary material for: The fibroblast Tiam1-osteopontin pathway modulates breast cancer invasion and metastasis
Source: Breast Cancer Res. 2016 Jan 28;18:14. doi: 10.1186/s13058-016-0674-8 (PMC4730665; doi:10.1186/s13058-016-0674-8)
Supplement: Additional file 2: — Supplemental figures S1-S8 and supplemental figure legends. (PDF 467 kb) [file 13058_2016_674_MOESM2_ESM.pdf]

Figure S1.

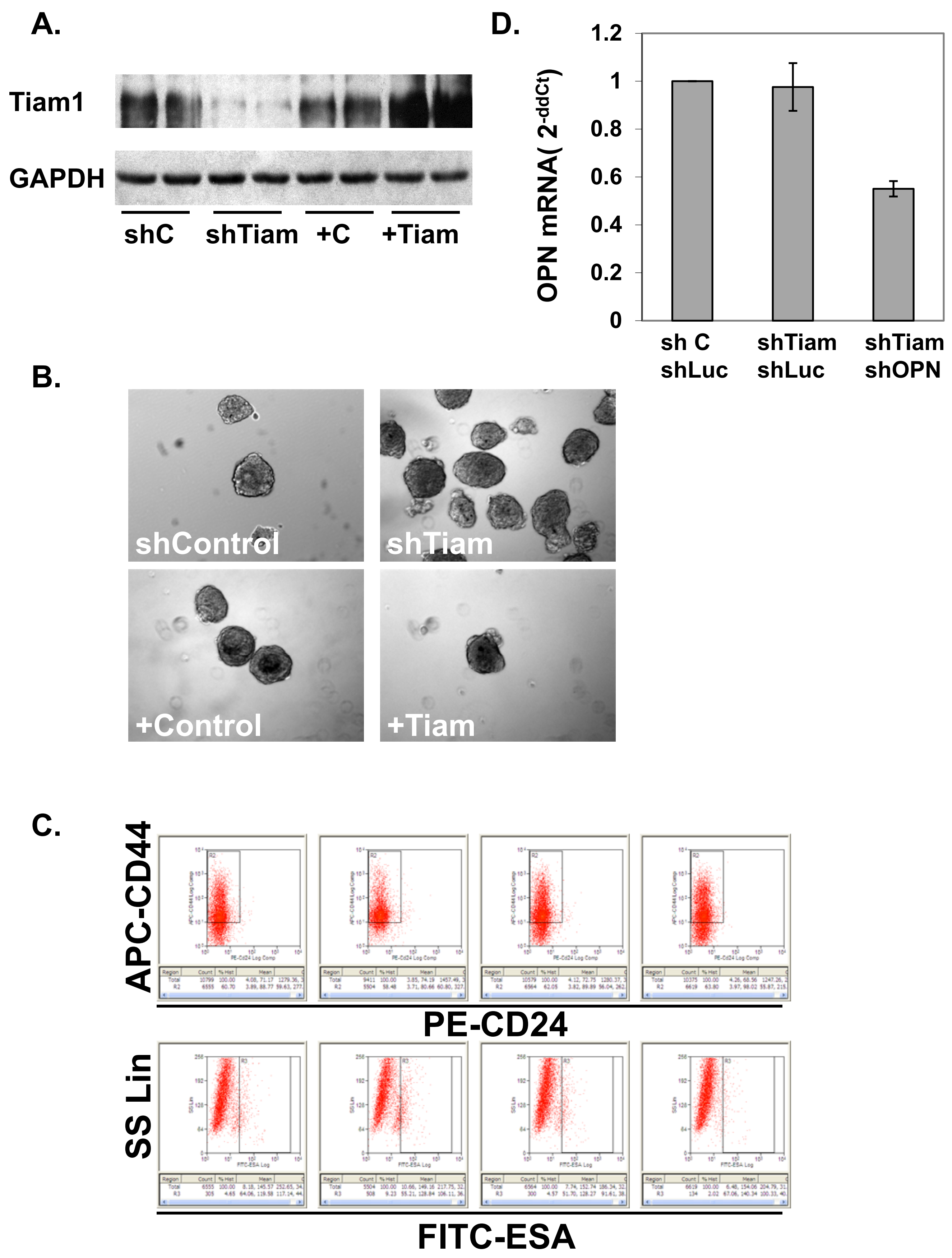
A.
Tiam1
GAPDH
shC
shTiam
+C
+Tiam
D.
OPN mRNA(2-ddCt)
1.2
1
0.8
0.6
0.4
0.2
0
sh C shLuc
shTiam shLuc
shTiam shOPN
B.
shControl
shTiam
+Control
+Tiam
C.
APC-CD44
PE-CD24
SS Lin
FITC-ESA

Figure S2.

A.

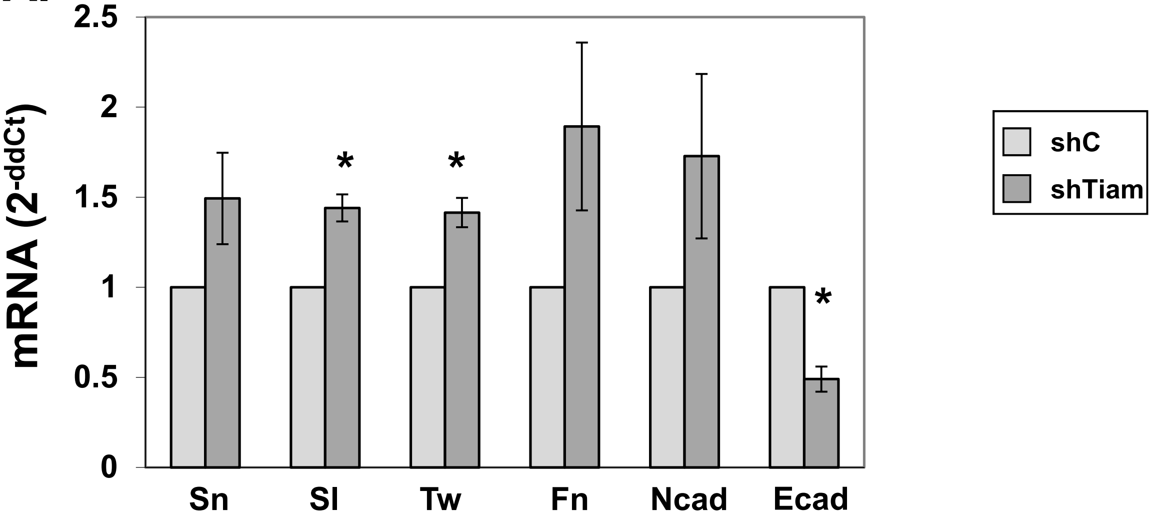
2.5
2
1.5
1
0.5
0
mRNA (2-ddCt)
*
*
*
Sn
Sl
Tw
Fn
Ncad
Ecad
shC
shTiam

B.

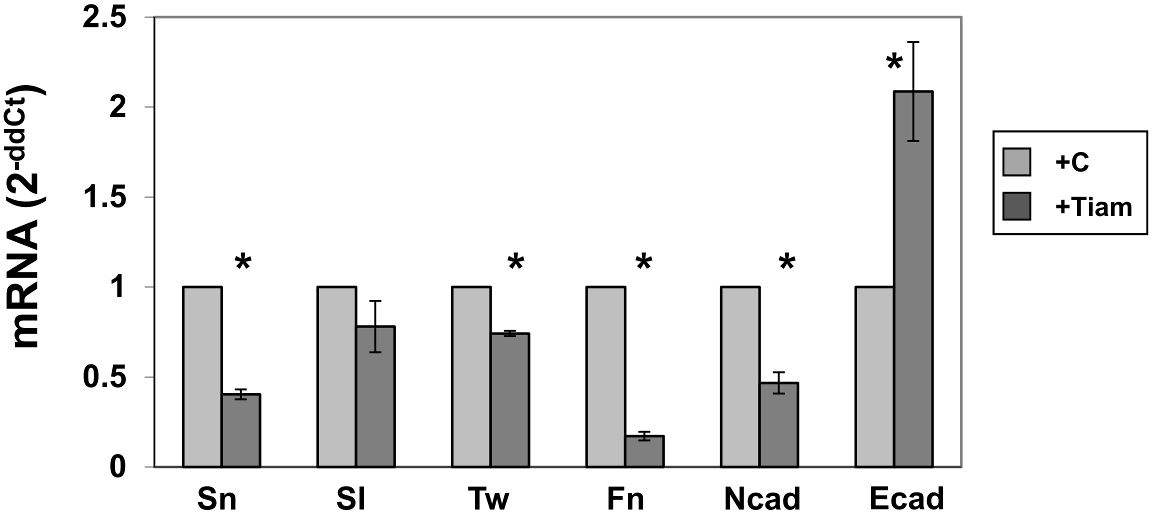
2.5
2
1.5
1
0.5
0
mRNA (2-ddCt)
*
*
*
*
*
Sn
Sl
Tw
Fn
Ncad
Ecad
+C
+Tiam

Figure S3.

A.

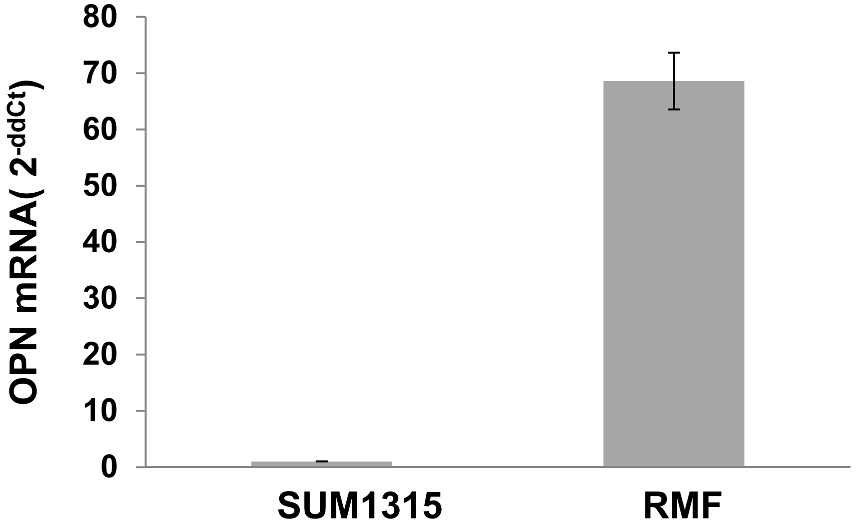

OPN mRNA(2^{-ddCt})
80
70
60
50
40
30
20
10
0
SUM1315
RMF

B.

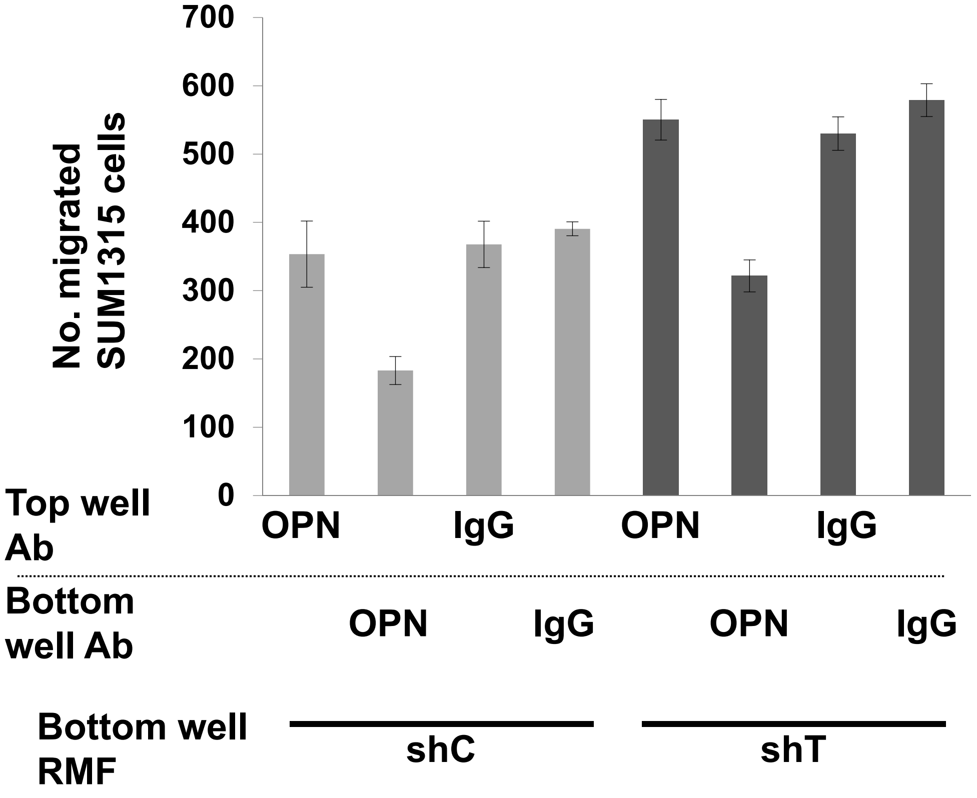

No. migrated SUM1315 cells
700
600
500
400
300
200
100
0
Top well Ab
OPN
IgG
OPN
IgG
Bottom well Ab
OPN
IgG
OPN
IgG
Bottom well RMF
shC
shT

Figure S4.

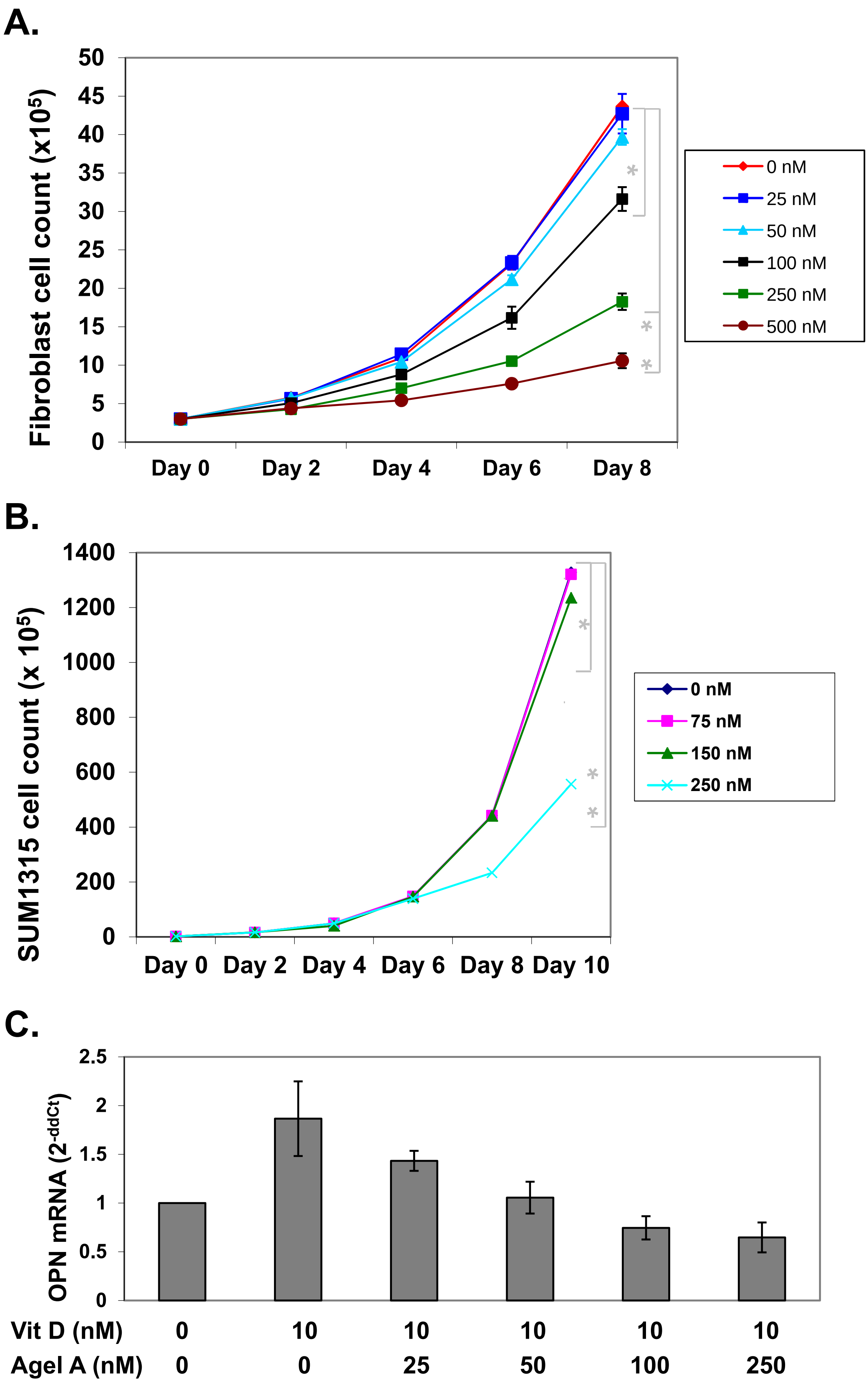
A.
Fibroblast cell count (x10^5)
50
45
40
35
30
25
20
15
10
5
0
Day 0
Day 2
Day 4
Day 6
Day 8
*
*
*
0 nM
25 nM
50 nM
100 nM
250 nM
500 nM
B.
SUM1315 cell count (x 10^5)
1400
1200
1000
800
600
400
200
0
Day 0
Day 2
Day 4
Day 6
Day 8
Day 10
*
*
*
0 nM
75 nM
150 nM
250 nM
C.
OPN mRNA (2^-ddCt)
2.5
2
1.5
1
0.5
0
Vit D (nM) 0 10 10 10 10 10
Agel A (nM) 0 0 25 50 100 250

Figure S5.

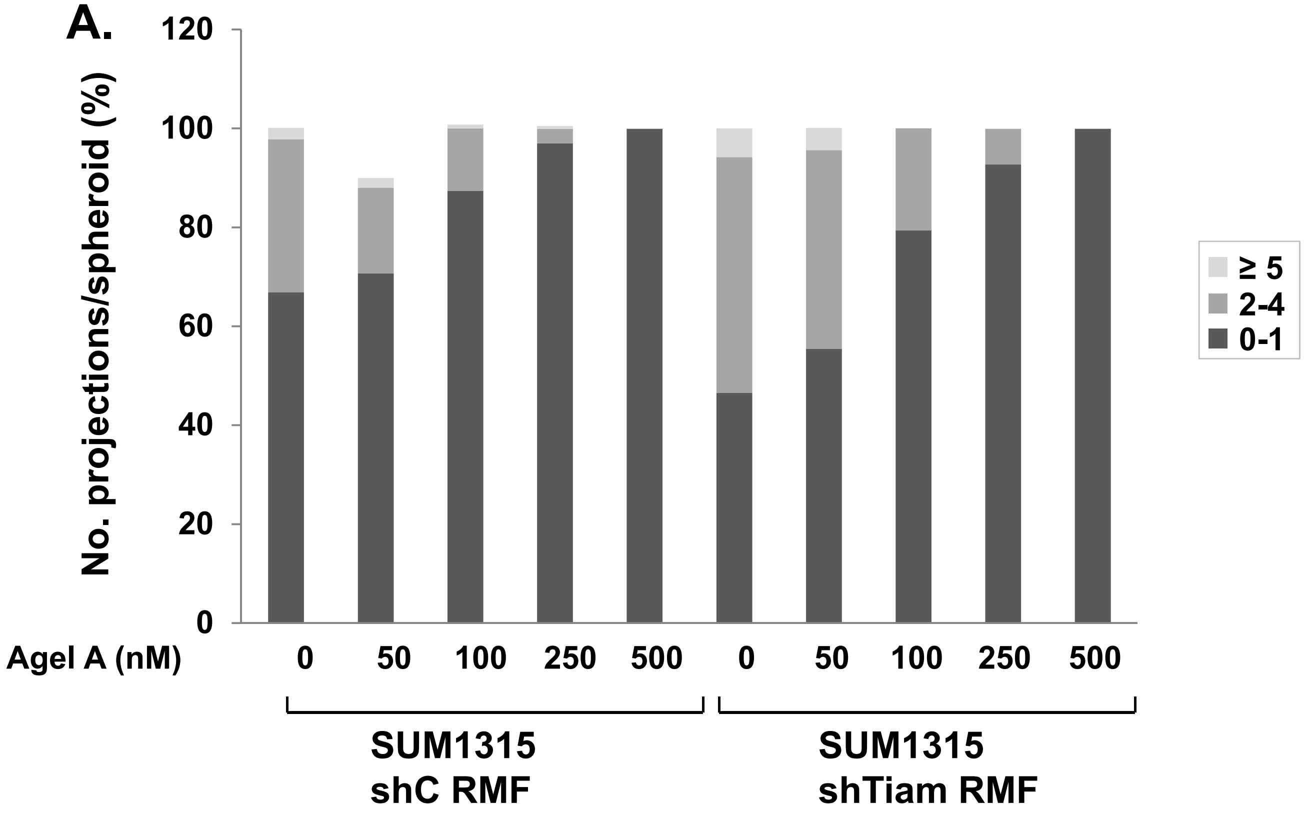
A.
120
100
80
60
40
20
0
No. projections/spheroid (%)
≥ 5
2-4
0-1
Agel A (nM)
0
50
100
250
500
0
50
100
250
500
SUM1315
shC RMF
SUM1315
shTiam RMF

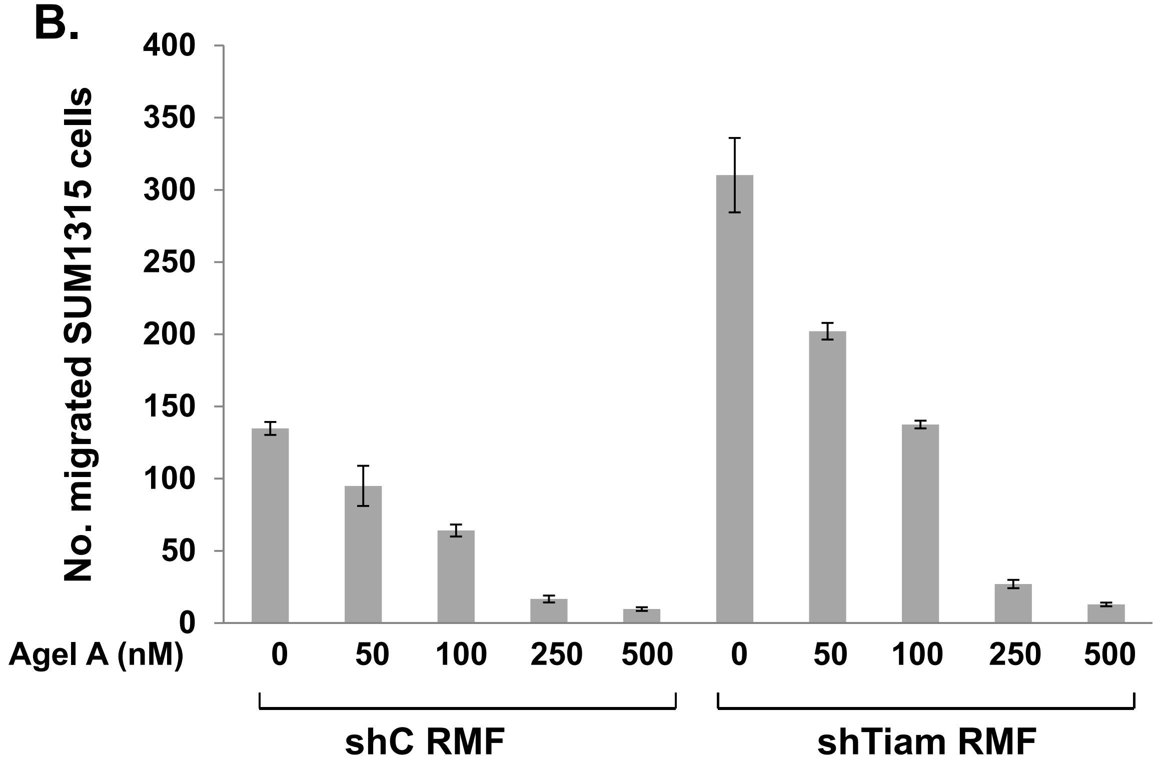
B.
400
350
300
250
200
150
100
50
0
No. migrated SUM1315 cells
Agel A (nM)
0
50
100
250
500
0
50
100
250
500
shC RMF
shTiam RMF

Figure S6.

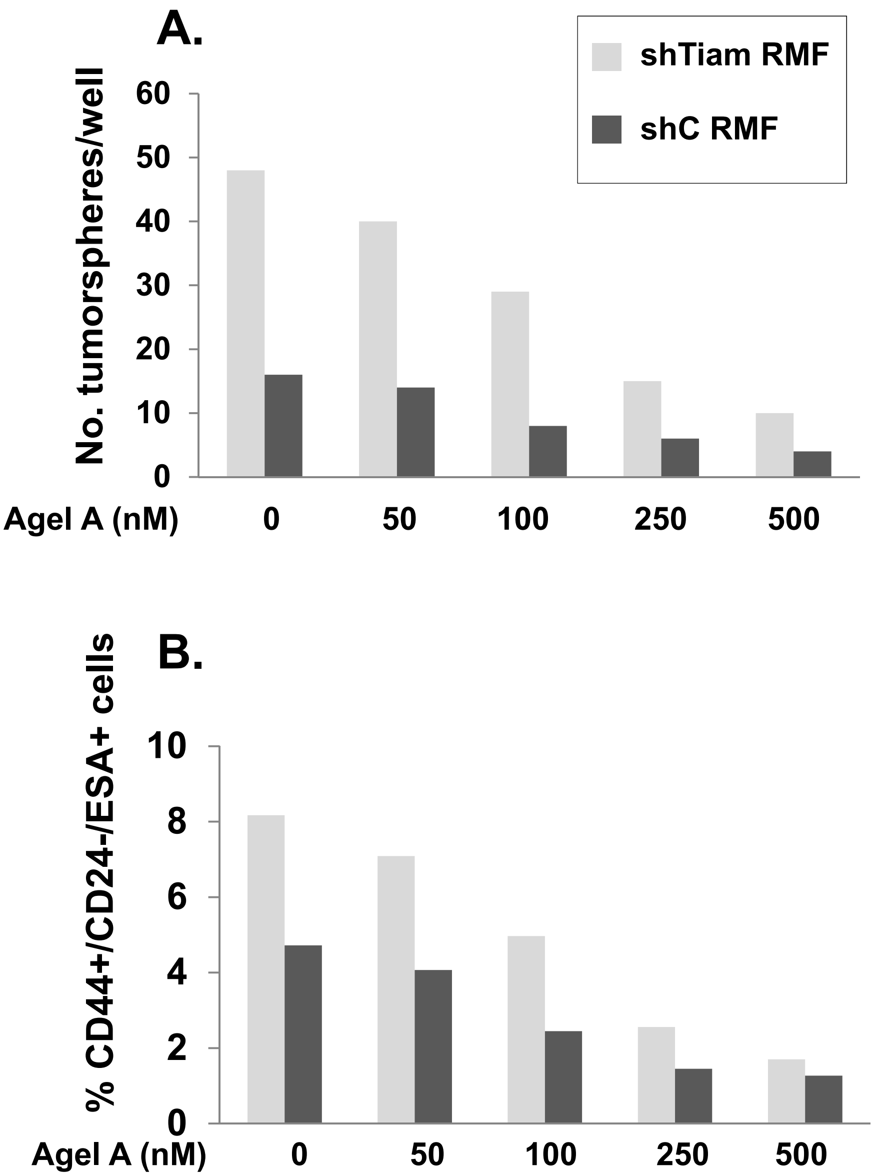

A.
shTiam RMF
shC RMF
No. tumorspheres/well
60
50
40
30
20
10
0
Agel A (nM)
0
50
100
250
500
B.
% CD44+/CD24-/ESA+ cells
10
8
6
4
2
0
Agel A (nM)
0
50
100
250
500

Figure S7.

A.

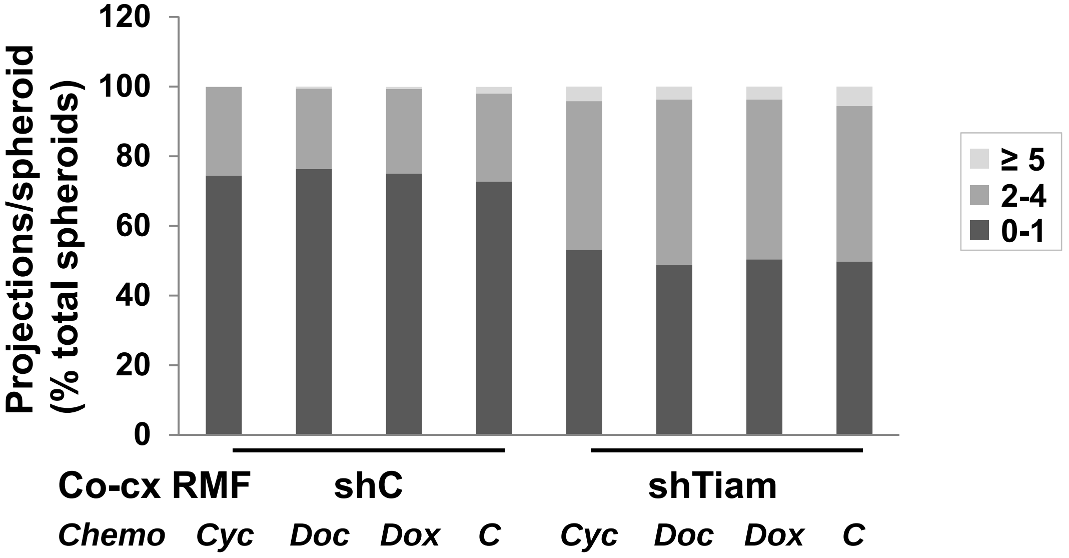

120
100
80
60
40
20
0
Projections/spheroid (% total spheroids)
≥ 5
2-4
0-1
Co-cx RMF
shC
shTiam
Chemo
Cyc
Doc
Dox
C
Cyc
Doc
Dox
C

B.

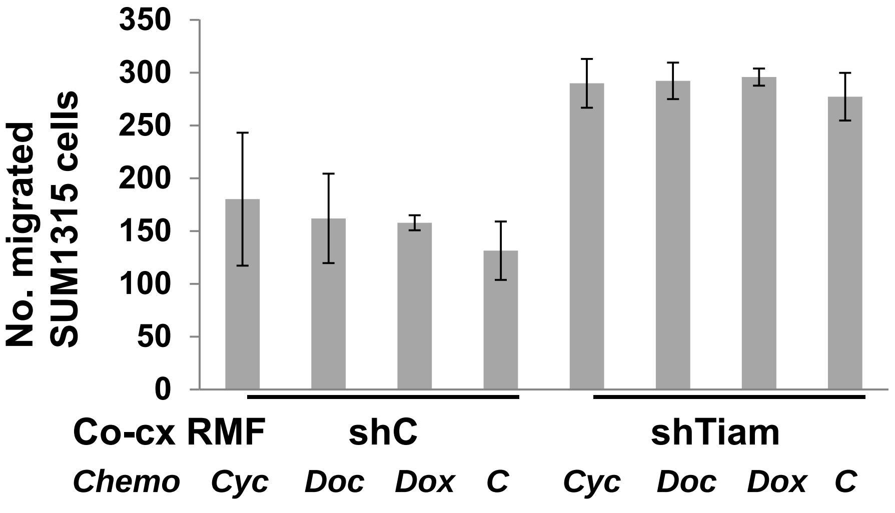

350
300
250
200
150
100
50
0
No. migrated SUM1315 cells
Co-cx RMF
shC
shTiam
Chemo
Cyc
Doc
Dox
C
Cyc
Doc
Dox
C

Figure S8.

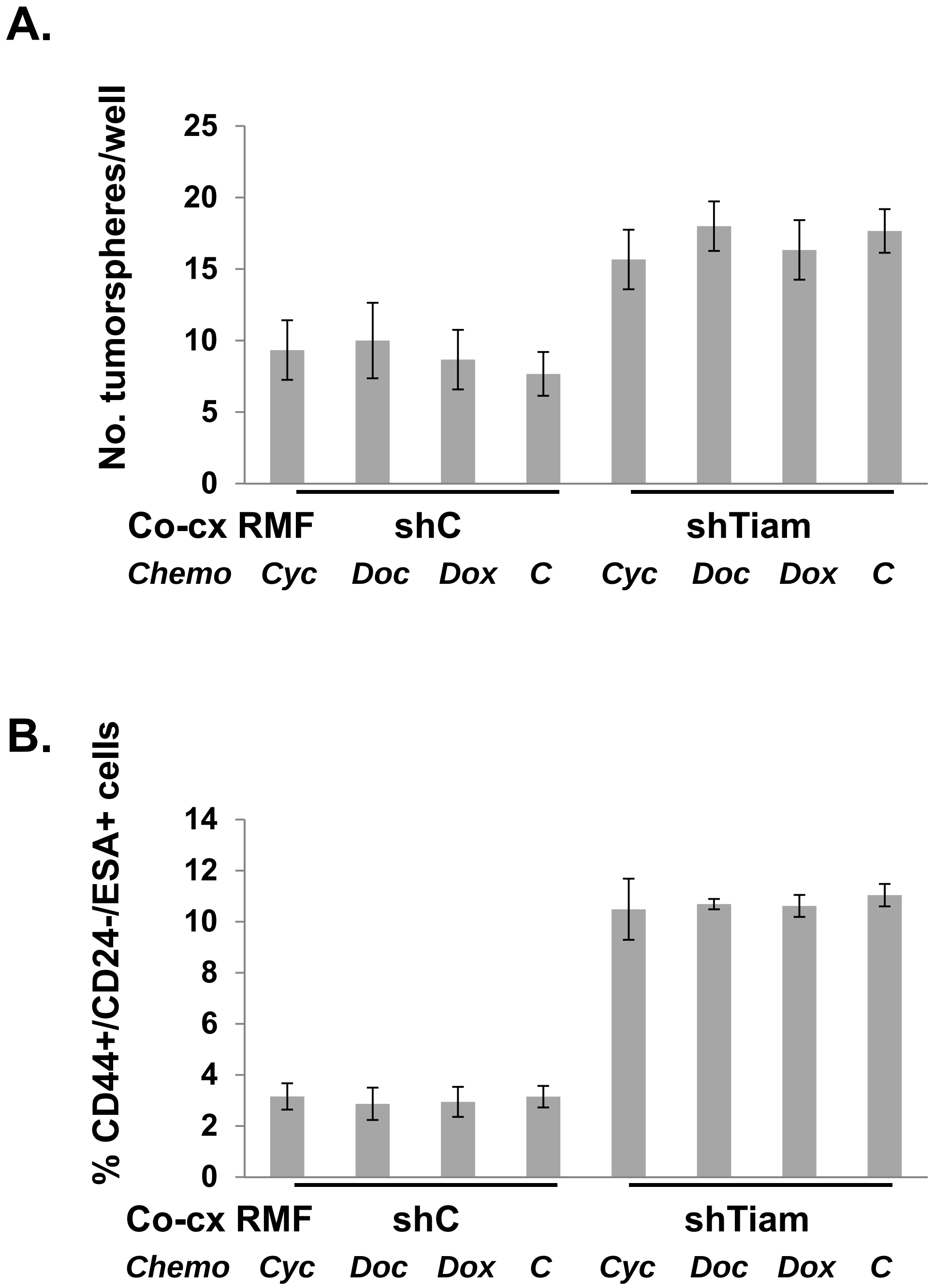
A.
No. tumorspheres/well
25
20
15
10
5
0
Co-cx RMF
shC
shTiam
Chemo
Cyc
Doc
Dox
C
Cyc
Doc
Dox
C
B.
% CD44+/CD24-/ESA+ cells
14
12
10
8
6
4
2
0
Co-cx RMF
shC
shTiam
Chemo
Cyc
Doc
Dox
C
Cyc
Doc
Dox
C

Supplemental Figure Legends

Fig S1. Verification of expression in engineered mammary fibroblasts, tumorsphere and flow cytometry subset analysis of post-co-culture SUM1315 cells.

A. Immunoblots from cleared cell lysates from control hairpin (shC), Tiam1-silencing hairpin (shTiam), pBabe control (+C), and pBabe-Tiam1-expressing (+Tiam) mammary fibroblasts, each loaded in duplicate lanes, incubated with Tiam1 antibody (top blot) or GAPDH antibody (bottom blot). B. Representative light microscope images of tumorspheres from SUM 1315 breast cancer cells isolated from mixed cell co-cultures with indicated mammary fibroblasts and quantitated as in figure 3A and 3B.

C. Representative flow cytometry data from SUM1315 breast cancer cells isolated from mixed cell co-cultures and assayed as in figure 3C and 3D. D. Quantitative RT-PCR for OPN mRNA from mammary fibroblasts containing control retroviral silencing hairpin and lentiviral luciferase silencing hairpin vectors (shC shLuc); Tiam1 and luciferase silencing hairpins (shTiam shLuc); Tiam1 and OPN silencing hairpins (shTiam shOPN). Results indicate mean +/- SD for triplicate samples.

Fig. S2. Effect of Tiam1 expression on EMT on SUM159 cells isolated from 3D co-cultures.

A. Quantitative RT-PCR for mRNA of indicated proteins relative to GAPDH control from SUM159 breast cancer cells isolated from 3D spheroid co-culture with control (shC) or Tiam1-deficient (shTiam) mammary fibroblasts. B. Quantitative RT-PCR for mRNA of indicated proteins from SUM159 breast cancer cells isolated from 3D spheroid co-culture with control (+C) or Tiam1-over-expressing (+Tiam) mammary fibroblasts. For A and B, results represent mean +/- SD for duplicate experiments; * indicates $p < 0.05$.

Fig. S3. Differential secretion of OPN from SUM1315 compared with RMF. A. Quantitative RT-PCR for osteopontin mRNA relative to GAPDH control from equal numbers of SUM1315 breast cancer cells and control RMF cells. B. Transwell migration of SUM1315 toward control (shC) or Tiam-deficient (shT) RMF seeded in bottom well. Some cells were treated with control IgG or OPN antibody before and during the assay (SUM1315 treated by indicated top well Ab; RMF treated by indicated bottom well Ab. Results represent mean cell counts +/- S.D. averaged across 9 high power fields for biologic triplicates.

Fig S4. Direct effects of Agelastatin treatment on cells in 2D culture. A and B. Cells were cultured in 2D with indicated concentrations of Agelastatin A. A. Mammary fibroblasts, mean +/- SD for triplicate wells. B. SUM1315 cells, values indicate single wells and are representative of duplicate experiments. * = $p > 0.2$; ** = $p < 0.001$ by Chi-square test. C. Quantitative RT-PCR for OPN mRNA from fibroblasts treated with 48 hours of vitamin D and Agelastatin A as indicated. Results indicate mean +/- SD and are representative of duplicate experiments, each in triplicate.

Fig S5. Agelastatin A effects on Tiam1-deficient fibroblast-mediated increase in invasion into matrix and migration of post-co-culture breast cancer cells. A. Number of projections/spheroid of mixed cell co-cultures with SUM1315 breast cancer cells and control or Tiam1-deficient fibroblasts incorporating Agelastatin A in indicated concentrations. Results indicated number of spheroids with indicated projections as percent of total spheroid population; at least 180 spheroids were counted for each condition. B. Transwell migration of SUM1315 after isolation from mixed cell spheroid co-cultures with control or Tiam1-deficient fibroblasts incorporating Agelastatin A in indicated concentrations. Cell counts were averaged across 9 high power fields for biologic triplicates.

Fig S6. Agelastatin A effects on Tiam1-deficient fibroblast-mediated increase in tumorsphere formation and CD44+/CD24-/ESA+ population in post-co-culture breast cancer cells.

A. Quantification of tumorspheres formed by SUM1315 cells after isolation from mixed cell spheroid co-cultures with control or Tiam1-deficient fibroblasts incorporating Agelastatin A in indicated concentrations. Results indicate mean for biologic triplicates. B. Flow cytometry quantification of sub-populations in SUM1315 cells after isolation from mixed cell spheroid co-cultures with control or Tiam1-deficient fibroblasts incorporating Agelastatin A in indicated concentrations.

Fig S7. Effects of chemotherapy on invasion and migration of SUM1315 cells in 3D co-cultures. A. Number of projections/spheroid of mixed cell co-cultures with SUM1315 breast cancer cells and control or Tiam1-deficient fibroblasts incorporating cyclophosphamide (Cyc), docetaxel (Doc), doxorubicin (Dox) or control media (C) as indicated. Results indicated number of spheroids with indicated projections as percent of total spheroid population; at least 150 spheroids were counted for each condition. B. Transwell migration of SUM1315 after isolation from mixed cell spheroid co-cultures with control or Tiam1-deficient fibroblasts incorporating indicated drug as in A. Cell counts were averaged across 9 high power fields for biologic triplicates.

Fig S8. Effects of chemotherapy on tumorsphere formation and cell sub-populations in post-co-culture SUM1315 cells. SUM1315 cells isolated from mixed cell spheroid co-cultures with control or Tiam1-deficient fibroblasts incorporating indicated drug as in Fig S10 were assayed for A. tumorsphere formation under ultra-low adherent conditions, and B. CD44+/CD24-/ESA+ sub-populations by flow cytometry. Results represent mean +/- SD for biologic triplicates.
