# Supplementary material for: The fibroblast Tiam1-osteopontin pathway modulates breast cancer invasion and metastasis
Source: Breast Cancer Res. 2016 Jan 28;18:14. doi: 10.1186/s13058-016-0674-8 (PMC4730665; doi:10.1186/s13058-016-0674-8)
Supplement: Additional file 3: — Supplemental Table S1. (DOCX 18 kb) [file 13058_2016_674_MOESM3_ESM.docx]

**Supplemental Table 1. Human breast cancer case summaries**

| **Unique Case Number** | **Surgical specimen tested** | **Age** | **Clinical info** | **Stage** |
| --- | --- | --- | --- | --- |
| 1 | Partial mastectomy | 62 | Inv ductal 2.2 cm Gr III, -LVI  Focal DCIS Gr III  ER 89, PR 33, HER2 3+ FISH equiv (1.8)  0/4 SLN | pT2 pN0(i-) |
| 2 | Core biopsy | 66 | Inv ductal 2.1 cm GrII, +perineural invasion  ER 96, PR 65, HER 1+  0/1 SLN | pT2N0 (i-) |
| 3 | Core biopsy,  Modified radical mastectomy | 57 | Inv ductal 3.5 cm GrIII  ER 0, PR 1, HER2 2+ FISH–  1/16 LN with ITC | pT2 pN0(i+) |
| 4 | Core biopsy | 63 | Inv ductal 1.9cm GrI, +LVI  DCIS 1 cm Gr I  Focal LCIS  ER 90, PR 15, HER 2+ FISH –  0/2 SLN | pT1c pN0 |
| 5 | Mastectomy | 60 | DCIS 12.5 cm Gr III  0/5 SLN | pTis (DCIS) pN0(i-) |
| 6 | Re-excision | 50 | Inv ductal 2mm Gr I  DCIS >2 cm Gr III  LCIS florid  ER 79, PR 60, HER2 1+  0/1 SLN | pT1a pN0(i-) |
| 7 | Core biopsy | 79 | Inv ductal 1.0 cm Gr I -LVI  DCIS 0.9 cm  ER 90, PR 80, HER2 2+ FISH-  0/3 SLN | pT1b pN0(i-) |
| 8 | Core biopsy,  Partial mastectomy | 61 | Inv papillary CA 1.3 cm Gr II -LVI  DCIS GrII 5% of tumor  ER 95, PR 80, HER2 1+  0/2 SLN | pT1c pN0(i-) |
| 9 | Excisional biopsy | 80 | Inv ductal 0.6cm Gr I,  ER>95, PR 1-9, HER2- | pT1b pNX |
| 10 | Partial mastectomy | 61 | DCIS 3 cm Gr I-II, multifocal  ER >95, PR 60 | pTis (DCIS) |
| 11 | Core biopsy | 59 | DCIS Gr I 3cm  ER >95, PR >95  0/4 SLN | pTis (DCIS) pN0(i-) |
| 12 | Core biopsy,  Partial mastectomy | 56 | DCIS 1.2 cm Gr III with microinvasion 0.1 cm  ER 90, PR 10  0/2 SLN | pT1mi pN0(i-) |
| 13 | Mastectomy | 60 | Inv ductal 0.5 cm Gr I -LVI  DCIS 2 cm Gr II  ER 95, PR 80, HER2 1+  0/3 SLN | pT1b pN0(i-) |
| 14 | Core biopsy | 76 | Inv ductal 0.9 cm Gr II -LVI  DCIS Gr I  ER 95, PR 95 HER2 0 | pT1b pN0(i-) |
| 15 | Simple mastectomy | 60 | Inv ductal 2 cm Gr I –LVI  Rare foci DCIS Gr II  ER 90, PR10, HER2 1+  0/1 SLN | pT1c pN0 (i-) |
| 16 | Core biopsy,  Partial MX | 53 | Inv ductal 0.9 cm Gr II –LVI  DCIS Gr II multifocal  ER 100, PR 90, HER2 0  0/1 SLN | pT1b pN0 (i-) |
| 17 | Partial mastectomy,  Total mastectomy | 80 | Inv ductal 4 cm GrII  DCIS 0.6 cm GrIII  ER 90, PR 20, HER2 1+  2/18 LN with extranodal extension | pT2 pN1a |
| 18 | Excisional biopsy,  Partial mastectomy | 62 | Inv ductal 3.1 cm Gr III, +LVI  Multifocal tumor with chest wall nodule  DCIS >3 cm Gr III  ER 95, PR 95, HER2 2+ FISH- | pT2 pN1a |
| 19 | Core biopsy,  Partial mastectomy | 68 | DCIS 3.6 cm Gr III  with micro-invasion 0.1 cm  ER 100, PR 0 HER2 0  0/1 SLN | pT1mi pN0 |
| 20 | Partial mastectomy | 47 | Inv CA with ductal + lobular features Gr I  DCIS 1.2 cm Gr II  ER 95, PR 95, HER2 0  2/12 LN | pT1c pN1a |
| 21 | Partial mastectomy | 52 | Microinvasion with  extensive DCIS 1.9 cm Gr III  ER 90, PR 0, HER2 0  0/2 SLN | pT1a pN0(i-) |
| 22 | Core biopsy | 68 | DCIS 2.8 cm Gr I  ER 95, PR 95  0/3 SLN | pTis (DCIS) pN0 |
| 23 | Partial mastectomy with reduction mammoplasty | 52 | Inv ductal 1.2 cm Gr I  Rare foci DCIS  ER 95,PR 95, HER2 1+ FISH-  0/3 SLN | pT1cN0 (i-) |
| 24 | Core biopsy  3cm palp mass  2cm mass on US  3.5 cm mass on MRI | 25 | Inv ductal Gr III  ER -, PR -  HER2- | cT2 NX |
| 25 | Re-excision | 59 | Inv ductal 1.25mm Gr I  DCIS >3.2cm cm Gr III  ER >90, PR 0-30  0/2 SLN | pT1a pN0 |
| 26 | Core biopsy | 37 | Inv ductal 1.0 cm Gr I, mult foci  High grade DCIS 4.5 cm  ER>95, PR 70  0/3 LN | pT1c pN0(i-) |
